# Playing in the clinical decision support sandbox: tools and training for all

**DOI:** 10.1093/jamiaopen/ooad038

**Published:** 2023-06-21

**Authors:** Andrey Soares, Majid Afshar, Chris Moesel, Michael A Grasso, Eric Pan, Anthony Solomonides, Joshua E Richardson, Eleanor Barone, Edwin A Lomotan, Lisa M Schilling

**Affiliations:** Division of General Internal Medicine, Department of Medicine, and the Data Science to Patient Value Initiative, School of Medicine, University of Colorado Anschutz Medical Campus, Aurora, Colorado, USA; Department of Medicine, School of Medicine and Public Health, University of Wisconsin Madison, Madison, Wisconsin, USA; Open Health Solutions Department, The MITRE Corporation, Bedford, Massachusetts, USA; University of Maryland School of Medicine, Baltimore, Maryland, USA; Westat Inc, Center for Healthcare Delivery Research and Evaluation, Rockville, Maryland, USA; Outcomes Research Network, Research Institute, NorthShore University HealthSystem, Evanston, Illinois, USA; Center for Health Informatics and Evidence Synthesis, RTI International, Chicago, Illinois, USA; Office of Health Informatics/Clinical Informatics and Data Management Organization, Veteran’s Affairs, Fayetteville, North Carolina, USA; Center for Evidence and Practice Improvement, Agency for Healthcare Research and Quality, Rockville, Maryland, USA; Division of General Internal Medicine, Department of Medicine, and the Data Science to Patient Value Initiative, School of Medicine, University of Colorado Anschutz Medical Campus, Aurora, Colorado, USA

**Keywords:** clinical decision support, FHIR, CQL, CDS Hooks

## Abstract

**Objectives:**

Introduce the CDS-Sandbox, a cloud-based virtual machine created to facilitate Clinical Decision Support (CDS) developers and implementers in the use of FHIR- and CQL-based open-source tools and technologies for building and testing CDS artifacts.

**Materials and Methods:**

The CDS-Sandbox includes components that enable workflows for authoring and testing CDS artifacts. Two workshops at the 2020 and 2021 AMIA Annual Symposia were conducted to demonstrate the use of the open-source CDS tools.

**Results:**

The CDS-Sandbox successfully integrated the use of open-source CDS tools. Both workshops were well attended. Participants demonstrated use and understanding of the workshop materials and provided positive feedback after the workshops.

**Discussion:**

The CDS-Sandbox and publicly available tutorial materials facilitated an understanding of the leading-edge open-source CDS infrastructure components.

**Conclusion:**

The CDS-Sandbox supports integrated use of the key CDS open-source tools that may be used to introduce CDS concepts and practice to the clinical informatics community.

## INTRODUCTION

Clinical Decision Support (CDS) is used by physicians and other healthcare professionals to improve health-related decisions and actions.^1^^,^^2^ Health care systems often independently design, build, and implement CDS rules using their vendors’ authoring and integration toolsets. Open-source resources that support the creation, sharing, and implementation of interoperable CDS artifacts can facilitate broader, standardized deployment of CDS. A CDS artifact is an implementation of a rule or related set of rules to guide specific evidence-based care (eg, reminding a clinician to prescribe statin for someone with a high ASCVD risk score). An ecosystem of shared CDS artifacts would contribute to broader and faster adoption of learning health systems (LHS) driven by “the seamless and rapid generation, processing, and practical application of the best available evidence for the circumstance.”^3^

In recent years, several health technology standards have emerged to address the need for reusable CDS knowledge artifacts. The Health Level Seven International (HL7^®^) Clinical Quality Language (CQL) standard,^4^ an author-friendly text-based language for developing machine-readable clinical logic, has gained traction in the CDS and electronic clinical quality measurement communities as evidenced by its incorporation into the Centers for Medicare & Medicaid Services (CMS) programs^5–8^ and the National Committee for Quality Assurance’s Healthcare Effectiveness Data and Information Set (HEDIS).^9^ The HL7 Fast Healthcare Interoperability Resources (FHIR^®^) standard, which defines formats and protocols for exchanging healthcare information electronically, has quickly been adopted by health technology companies across the world.^10^ Other relevant standards have arisen out of FHIR, such as HL7 CDS Hooks for invoking CDS services^11^ and Substitutable Medical Applications and Reusable Technologies (SMART) on FHIR for integrating FHIR-enabled apps.^12^

In 2018, CQL was adopted by the CMS to represent electronic clinical quality measures (eCQMs)^5–8^ which are used in US National CMS quality measure programs such as the Merit-Incentive Payment System^7^ and the Medicare Shared Savings Programs.^8^ The Office of the National Coordinator for Health Information Technology (ONC) and the Centers for Disease Control (CDC) partnered to create the CDC’s Opioid Prescribing Electronic Clinical Decision Support tools, using CDS Hooks, CQL, and FHIR standards, such that they would be implementable within any electronic health record (EHR) system.^13^ Results from a 2021 survey specific to CDS use,^14^ found that these tools were useful for CDS, that uptake was increasing, but use in real-world environments beyond pilots remains low. The Agency for Healthcare Research and Quality (AHRQ) and others have released publicly available and reusable CDS artifacts and tools (eg, CDS Authoring Tool and CQL Services) to accelerate the adoption of these standards for use in CDS.^15^ Unfortunately, standards-based CDS artifacts and tools are still not widely adopted. CDS developers and implementers need an open platform for education and dissemination of resources that seamlessly introduces these many CDS-related standards and tools to the clinical informatics community, as well as a platform for creating and testing CDS.

## OBJECTIVES

We introduce the CDS-Sandbox, a cloud-based platform created to offer hands-on experience using key FHIR- and CQL-based open-source tools and technologies for building, testing, and implementing reusable CDS artifacts. The goal of the CDS-Sandbox is to integrate various CDS components for more efficient creation and testing of CDS applications and to help potential users gain literacy on how the components function together in the development and support of CDS systems. We demonstrate a test case of the CDS-Sandbox, and describe user feedback of a workshop tutorial we designed for using the CDS-Sandbox that was conducted at the 2020 and 2021 American Medical Informatics Association (AMIA) Annual Symposia.^16^

## MATERIALS AND METHODS

The CDS-Sandbox contain components that enabled workflows for authoring, testing, and implementing CDS artifacts (Table 1). A user could use the CDS-Sandbox to create a sample CDS artifact, starting from a set of evidence-based guidelines, translating the artifact into a machine-readable syntax representation, testing the translated artifact against synthetic patient data from a demo FHIR server, and triggering the artifact from a simulated EHR environment. We used CQL to write the CDS logic and FHIR R4 to support data interoperability. The CDS-Sandbox used in the 2021 workshop was hosted on Amazon Web Services (AWS) instances configured with 2 CPUs, 8GB Memory, 30GB Disk, and Ubuntu Linux. To access the CDS-Sandbox on AWS, users needed a remote desktop application, such as Microsoft Remote Desktop, installed on their computers, and a Unified Medical Language System (UMLS)^17^ account and Application Programming Interface (API) key to access the Value Set Authoring Center (VSAC).^18^ Access to VSAC enabled the CDS Authoring Tool, CQL Results SMART on FHIR app, and CQL Services to find and download value sets used by the CQL libraries.

**Table 1.** Description of the main tools in the CDS-Sandbox

Tools	Functional description
CDS Authoring Tool	The AHRQ CDS Connect CDS Authoring Tool allows users to author CDS logic using a simple web-based interface and to export it as FHIR-based CQL libraries.
CQL Services	CQL Services, also developed as part of the AHRQ CDS Connect project, enables integrators to easily expose CQL logic libraries over a CDS Hooks API.
CDS Hooks Sandbox	The CDS Hooks Sandbox provides authors and integrators a way to test CDS Hooks API services using synthetic patient data.
CQL-to-ELM Translator	The CQL-to-ELM translator converts CDS artifacts from the CQL format into an expression logical model (ELM) format that is easier for other applications to process.
SMART on FHIR Launcher	The SMART on FHIR Launcher provides another approach for testing by allowing users to launch the CQL Results app and access synthetic patient data from the FHIR server.
CQL Results app	A SMART on FHIR app was created to process the CQL libraries against synthetic patient data and display the results of the logic.
HAPI FHIR server	The HAPI FHIR Server contains synthetic patient data, created with Synthea, that is used for testing the artifacts. It provides services over a standard FHIR interface.
VSAC	Several services rely on the National Library of Medicine’s (NLM) Value Set Authority Center (VSAC) for searching and downloading value sets. The VSAC is the only service that is used inside the virtual machine (VM) but hosted outside of the VM.

Prior to the workshop, we successfully completed beta testing of the tutorial and CDS-Sandbox tools with members of our development team and invited informaticians. Testers followed the step-by-step tutorial instructions and provided feedback on the content, usability, and time for completing the tutorial. After multiple iterations, all users across different levels of expertise completed the full tutorial in under 120 min, which was the time allotted during the workshop.

The CDS-Sandbox was used to conduct a workshop at 2 AMIA Annual Symposia. For the 2020 version (virtual), participants were required to download and install the CDS-Sandbox on their computers. For the 2021 version (in-person), participants accessed the CDS-Sandbox in the cloud. The learning scenario of the workshop tutorial was a simplified version of AHRQ’s CDS artifact “Statin Use for the Primary Prevention of CVD in Adults: Clinician-Facing CDS Intervention,” which was freely available on the CDS Connect repository.^19^ Our guideline was a statin therapy recommendation for adults aged 40 to 75 years who had one or more CVD risk factors (ie, LDL>130 mg/dL or hypertension) and a calculated 10-year CVD event risk score of 10% or greater and were not currently on statin therapy. We also leveraged the complete CDS Connect artifact “CMS’s Million Hearts Model Longitudinal ASCVD Risk Assessment Tool for Baseline 10-Year ASCVD Risk” to demonstrate the reuse of an existing artifact to support the creation of new artifacts.^20^ To test the logic of the Statin artifact, we created synthetic patient data with specific health history to represent patients that received a Statin recommendation, as well as patients who would not.

**Figure 1.**
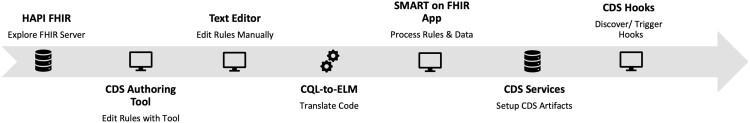
Steps used in the tutorial to demonstrate the CDS-Sandbox components.

**Figure 2.**
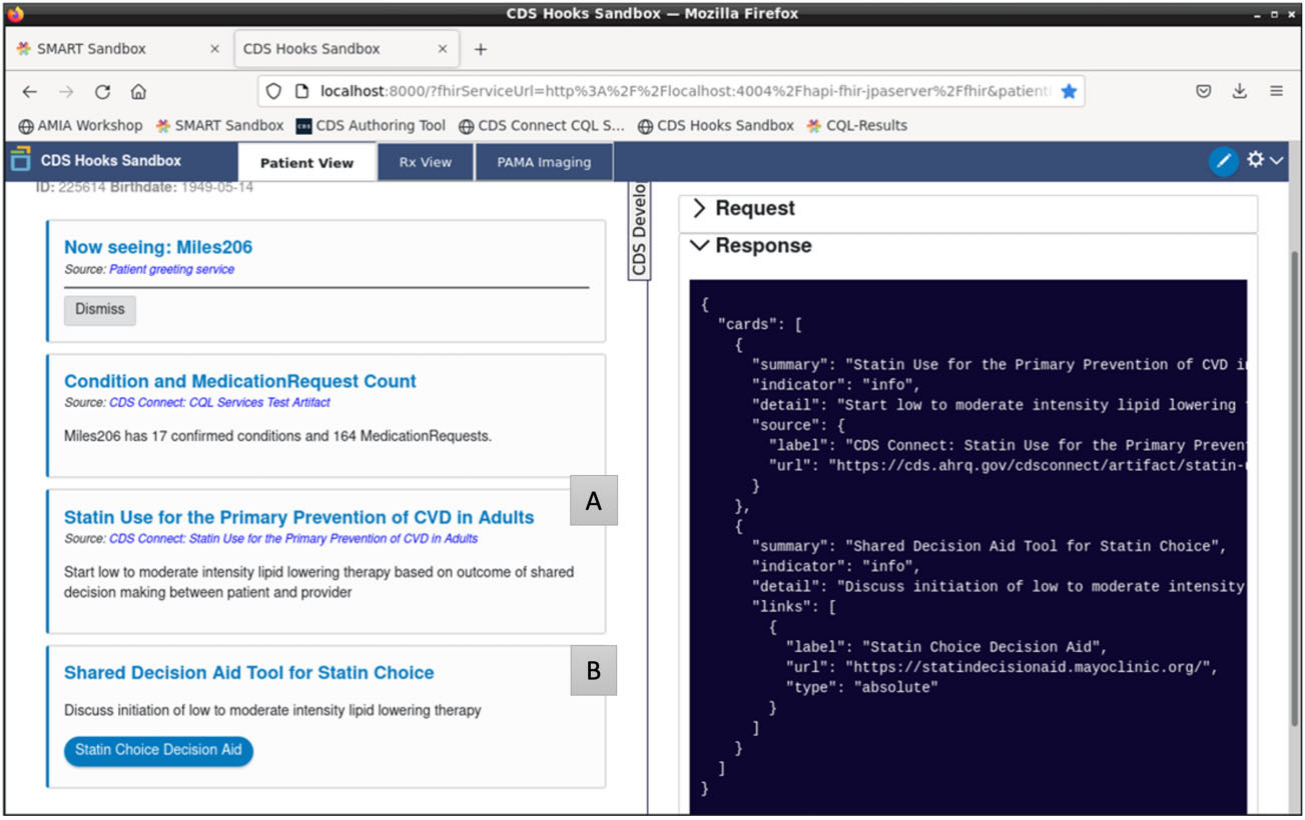
Sample triggering of CDS Hooks cards with (A) Statin recommendation and (B) link to a Statin Decision Aid tool.

In the tutorial we provided a guide with explanation, instruction, and hands-on use of all the CDS-Sandbox components including steps (Figure 1) for viewing patients available for testing in the HAPI FHIR Server, creating new CQL artifacts using AHRQ’s CDS Authoring Tool, editing CQL artifacts manually, translating CQL to Expression Logical Model (ELM), launching a SMART on FHIR app that could process patient data against the CQL artifact, loading the created artifact in AHRQ’s CQL Services and setting up a CDS Hooks service, and using CDS Hooks to discover and trigger the Statin artifact and present the Statin recommendation. In the final step, participants experienced the CDS Hooks being triggered with the statin recommendation and a Statin Decision Aid tool hyperlink displayed (Figure 2).

The tutorial workshop was 120 minutes, which allowed us to guide participants through completion of the 7 steps of the hands-on portion of the workshop. To support a variety of attendees with varying familiarity with the tools and to support participants who fell behind, we created scripts with rescue-files and skip steps so that participants could optionally skip steps for any reason, such as not having a UMLS account, not being familiar with a specific tool, not having interest in writing code manually, or trying to catch up. In addition, we included scripts to facilitate creating folders and copying files for participants who were not familiar with performing those tasks in a Linux environment. Instructions to build the CDS-Sandbox and a copy of the tutorial materials used in the workshop were open-source and publicly available (see Data Availability). The full workshop tutorial guide with step-by-step instruction is available in the Supplementary Material.

## RESULTS

To test the feasibility and learnability of the CDS-Sandbox and tutorial materials, we piloted them at 2 AMIA Symposium workshops. Both workshops were well attended, including 84 virtual participants in 2020 and 31 in-person participants in 2021. Feedback from the first virtual workshop was positive for its technical content but less so for the prerequisites and pace of the tutorial. These observations informed modifications and improvements to the CDS-Sandbox environment and the CDS-Sandbox tutorial guide (see Supplementary Material). In 2021, we provided preconfigured CDS-Sandbox instances and the face-to-face format allowed us to facilitate hands-on support during the workshop.

In 2021, 9 of 31 participants provided feedback to an online survey after the workshop. Among the respondents there were 4 developers or informaticists, 2 administrators, 1 clinician, 1 educator, and 1 student across the following work settings: private sector (*n* = 4); academia (*n* = 3); and other (*n* = 2). No one reported having extensive prior experience with the subject matter; 2 had moderate experience, 6 had limited experience, and 1 had no experience. All 9 participants reported that it was either somewhat or very easy to perform the prerequisites (range of 4.0–5.0 on a 5-point Likert scale). The majority reported being “very satisfied” with the workshop (Table 2). Participants reported they would use the knowledge they gained to implement or integrate something into a development environment (*n* = 5), implement or integrate something into an academic environment or clinical environment (*n* = 3), and/or further contribute to the development of these or related standards-based tools (*n* = 3). Despite the high levels of satisfaction, 4 reported being “very uninterested” if additional workshops like this one were offered, while 5 reported being interested.

**Table 2.** Workshop satisfaction with the workshop and interest in additional workshops (*n* = 9)

	Very satisfied	Somewhat satisfied	Neither satisfied nor dissatisfied	Somewhat dissatisfied	Very dissatisfied
What was your satisfaction with aspects of this workshop?					
Content	8	1	0	0	0
Duration	8	1	0	0	0
Format	7	1	1	0	0
How interested would you be in additional workshops like this from the AMIA CDS Workgroup?					
	4	0	0	2	3

Lastly, 8 participants provided free text feedback with the following comments: (1) “A wonderful, eye-opening session where the hands-on components really teach a lot. Enjoyed it immensely!” and (2) “I really appreciate all the advance work that the team put into making this workshop available!” One participant expressed, “a larger discussion on the challenges…with concept sets and logic (eg, how to select proper codes for inclusion/exclusion criteria, or how to deal with patients with missing data).”

## DISCUSSION

We have showcased the CDS-Sandbox as a workshop both virtually and in-person with positive responses from participants. The most recent version of the CDS-Sandbox has incorporated feedback and lessons learned from the AMIA workshops. We believe the current version is suitable for both informatics and clinical audiences to learn how to develop and use knowledge artifacts and CDS services. The CDS-Sandbox and tutorial materials may be disseminated into education programs, training seminars, and research activities.

The long-term goal of the CDS-Sandbox and tutorial is to facilitate and reduce barriers in access to CDS solutions and move towards a national CDS repository for all healthcare organization and systems.^21^ Training in workshops similar to ours is intended to increase familiarity with CQL and standards-based authoring of CDS, help clinical informaticians become more comfortable with direct authoring of CDS artifacts, and create a community open to sharing and reusing CDS artifacts. A recent publication described a systematic process for translating a clinical pathway into CQL-based CDS artifacts.^22^ However, our work goes further by showing how to build upon existing artifacts as well as develop new ones, test the artifact, and deploy the artifact in a simulated EHR environment. The CDS-Sandbox is an end-to-end translation from artifact development to deployment and the first of its kind for training and education to support the creation and sharing of CDS artifacts.

Several limitations exist in the uptake of standards in CDS, including FHIR, SMART, CQL, and CDS Hooks. Many standards have not become ubiquitous in real-world practice and remain largely in pilot programs. Much of the evidence on usage only began surfacing after 2018 and few pilot programs were using CQL and CDS Hooks.^17^ Most CDS implementation and effectiveness studies that used standards-based tools for CDS development remain early stage, which suggests a need for better dissemination of standards-based CDS tools and artifacts like our open-source tutorial guide.^14^^,^^23^ Large health care systems with ample resources still build and implement CDS with vendor supported tools, but uptake of standards in CDS is gradually increasing with more regulations and impetus by the federal government to foster wider interoperability through the Office of the National Coordinator’s Final Rule and the 21st Century Cures Act.

## CONCLUSION

The CDS-Sandbox introduces several critical components necessary for the creation of shareable and interoperable CDS artifacts and systems. These elements have been harnessed together in a VM with tutorial instructions that support integrated use of the key CDS tools, allowing users to begin with a narrative clinical guideline and create a sharable CDS artifact. Our open-source tutorial may be used to introduce and practice CDS concepts for beginners but also guide developers to use the components with much greater complexity for creating interoperable CDS.

## Supplementary Material

ooad038_Supplementary_Data

## Data Availability

The GitHub page contains instructions to build the CDS-Sandbox described in this study (https://github.com/CUD2V/cds-sandbox-workshop), and a copy of the tutorial material used for the hands-on exercise during the AMIA 2021 workshop is available as Supplementary Material.
