# Supplementary material for: Playing in the clinical decision support sandbox: tools and training for all
Source: JAMIA Open. 2023 Jun 21;6(2):ooad038. doi: 10.1093/jamiaopen/ooad038 (PMC10283349; doi:10.1093/jamiaopen/ooad038)

AMIA 2021 Annual Symposium

Workshop 13:

Open-Source and Interoperable Approaches for Clinical Decision Support (sponsored by Clinical Decision Support Working Group)

Andrey Soares[1]; Majid Afshar*[2]; Joshua E. Richardson*[3]; Chris Moesel[4]; Anthony Solomonides,[5]; Eleanor Barone*[6]; Eric Pan*[7]; Michael A. Grasso[8]; Lisa Schilling[1]; on behalf of the AMIA Clinical Decision Support Working Group*

[1]University of Colorado School of Medicine, Aurora, Colorado; [2]University of Wisconsin School of Medicine and Public Health, Madison, WI; [4]MITRE Corporation, Maclean, VA; [5]Northshore University, Evanston, IL; [6]Veterans Administration Medical Center, Fayetteville, NC; [7]Westat, Rockville, MD; [8]University of Maryland, Baltimore, MD

Part II - Interactive Content Delivery: CDS Hands-On Simulation

---

Prerequisite Duration:

~3-5 business days to obtain the UMLS account and API Key
~0.5 hour to setup the Remote Desktop application

Tutorial Duration:

~120 minutes to complete the steps

Led by:

Andrey Soares

Educational Objectives:

- Acquire a comprehensive knowledge of existing open-source CDS tools and resources.
- Be able to author CDS rules (aka artifacts) in a test environment.
- Understand the advantages and disadvantages of the different publicly available CDS tools
- Run a CDS tool from end-to-end.

Prerequisites:

- Register for a **Unified Medical Language System (UMLS)** Account. It might take 3-5 business days to get approval.
- Install a Remote Desktop application to access the Amazon Web Services (AWS) instance (henceforth, AWS VM)
- Review the CDS Authoring Tool User Guide: https://cds.ahrq.gov/authoring/documentation

Acknowledgements:

- HL7®, and FHIR® are the registered trademarks of Health Level Seven International and their use of these trademarks does not constitute an endorsement by HL7.
- We would like to thank the organizations and contributors to the technologies and open-source tools used in this tutorial.
    - AHRQ
    - Clinical Quality Framework
    - HAPI FHIR
    - HL7
    - MITRE Corporation
    - SMART Health IT

Disclosure:

- The content in this tutorial is up to date as of October 2021 and is derived directly from open-source materials. The tools are presented "As is". Please refer to the original software repositories and documentation for updates and version control. The materials and content displayed in this workshop do not reflect the official policy or position of any agency, organization, or employer. The authors do not accept any responsibility for any of the source materials and software. The AWS Image contains synthetic patient data.
  Do not upload Personally Identifiable Information (PII) or Protected Health Information (PHI).

OVERVIEW

Part II of the workshop will provide participants with hands-on experience using several open-source Clinical Decision Support (CDS) tools and resources, including CDS Connect, CDS Hooks, and SMART on FHIR. The learning scenario will be statin use in at-risk adults. Participants will work in a FHIR-based open-source CDS Sandbox environment hosted on AWS. We will use Clinical Quality Language (CQL) to write the CDS logic and use FHIR R4 for data interoperability.

The CDS artifacts used in this tutorial are adapted from CDS Connect's CDS artifacts "Statin Use for the Primary Prevention of CVD in Adults: Clinician-Facing CDS Intervention" and the "CMS's Million Hearts® Model Longitudinal ASCVD Risk Assessment Tool for Updated 10-Year ASCVD Risk". The original artifacts, created for FHIR DSTU2 and FHIR R4, are freely available from AHRQ's CDS Connect website.

The tutorial includes steps for: (1) viewing patients available for testing in the HAPI FHIR Server, (2) creating new CQL artifacts using AHRQ's CDS Authoring Tool (CAT), (3) editing CQL artifacts manually, using a text editor, (4) translating CQL to Expression Logical Model (ELM), (5) launching a SMART on FHIR app that can process patient data against the CQL artifact and present the results on the screen, (6) loading the artifact created to the AHRQ's CQL Services and setup a CDS Hook service, and (7) discovering and triggering CDS Hooks to demonstrate the CDS created.

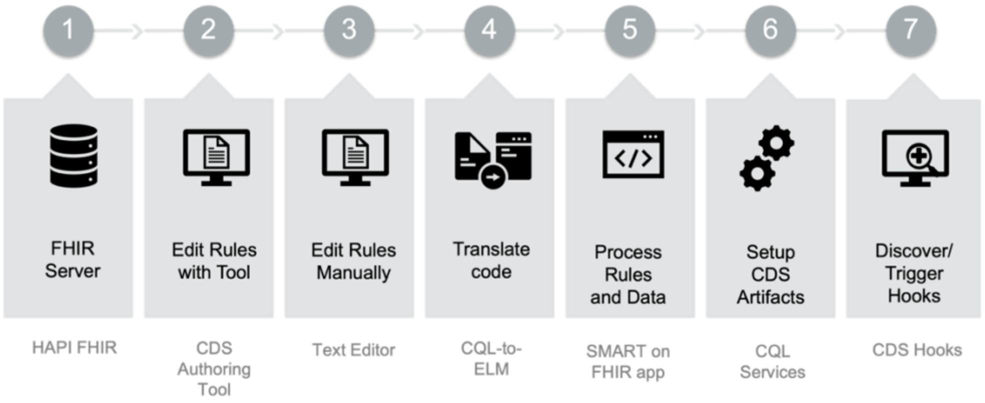

You must have a **valid UMLS account/API KEY** and **access to the Remote Desktop** to successfully complete this tutorial.

CDS Sandbox VM

The AWS VM contains several services available on localhost (using specific ports) and tools to support the creation of CQL artifacts. The services will be accessible via the AWS VM's internet browser (Firefox). The Value Set Authority Center (VSAC) is accessed for three parts of the tutorial: (1) by the CDS Authoring Tool to search value sets and codes, (2) by the CQL-Results app to update the value set database, and (3) by the CDS Services to download the value sets to be used by the CQL libraries.

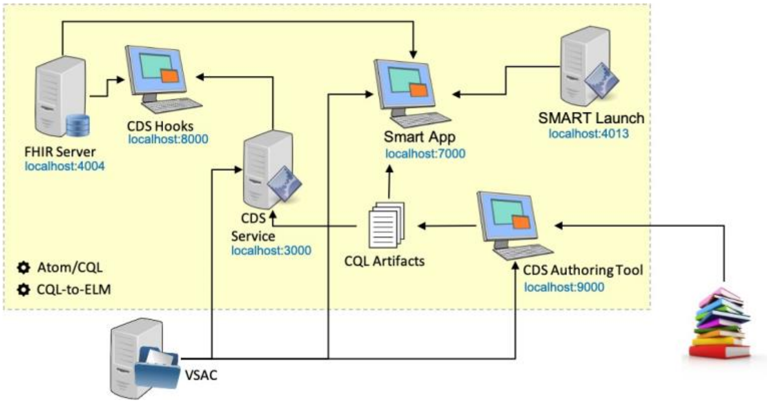

Statin Use

For this tutorial we will create a CDS artifact for Statin Therapy. The logic is based on the **Statin Use for the Primary Prevention of CVD in Adults: Clinician-Facing CDS Intervention** artifact from the CDS Connect artifacts repository (https://cds.ahrq.gov/cdsconnect/repository). The artifact was developed and piloted for FHIR DSTU2 by the MITRE Corporation and updated to FHIR R4 in 2021. It presents a United States Preventive Services Task Force (USPSTF) statin therapy recommendation for adults aged 40 to 75 years without a history of cardiovascular disease (CVD) who have 1 or more CVD risk factors (i.e., diabetes, hypertension, or smoking) and a calculated 10-year CVD event risk score of 10% or greater. Below is a summary of the *partial* guidelines for Statin chosen specifically for this tutorial.

Inclusion

- Age 40 to 75
- AND One or More Risk Factors (Group)
    - LDL > 130 mb/dL
    - OR Has Hypertension

Exclusion

- On Satin Therapy (Medication Request)

Recommendation

- Start low to moderate intensity lipid lowering therapy based on outcome of shared decision making between patient and provider

⚠ This tutorial is for demonstration purposes only. It uses partial guidelines for Statin and is **not clinically complete**. You should not use this sample with any patient.

We will complete the CQL artifact in two steps:

1. Use the CDS Authoring Tool workspace to import external CQL libraries, create inclusion criteria, and define the recommendations.
2. Use a text editor to modify CQL code manually by adding new inclusion and exclusion criteria, and translate the CQL file to ELM (JSON file) to be used by the CQL Services and SMART on FHIR app.

PREREQUISITES

Install Remote Desktop to access the AWS Image

Download and install a remote desktop client application such as **Microsoft Remote Desktop**. Mac users can get it from Apple Store.

- Click on the Plus Sign and select **Add PC**

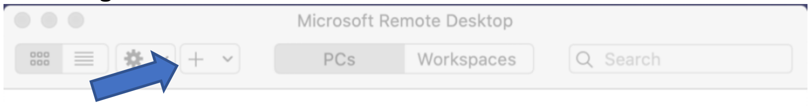

- Enter the **IP Address** (USE YOURS!!!) you received for this workshop in the **PC name** field.
  NOTE: Each workshop participant will have a unique IP address.

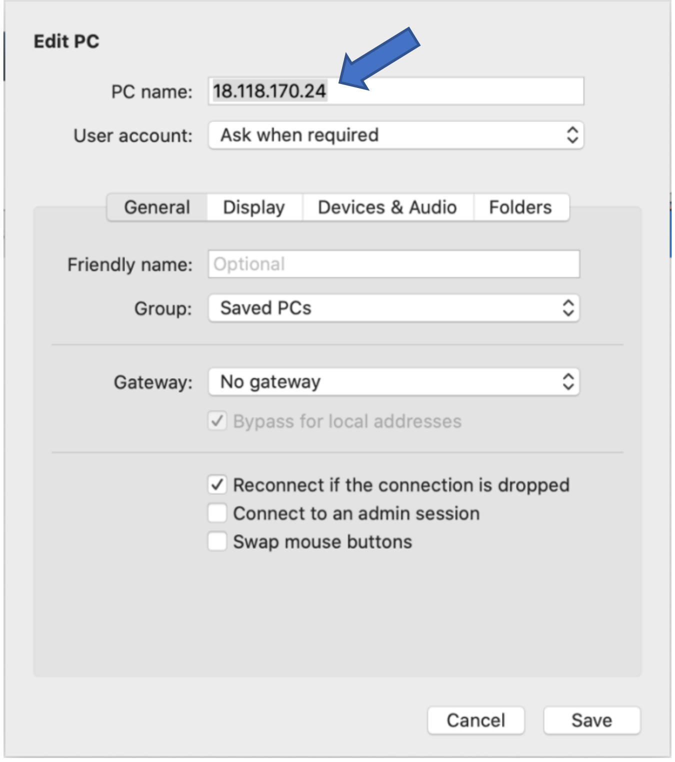

- In the Display tab:
  - Uncheck the option Start session in full screen
  - Check the option Fit session to window.
  - Click **Save** to complete the setup.

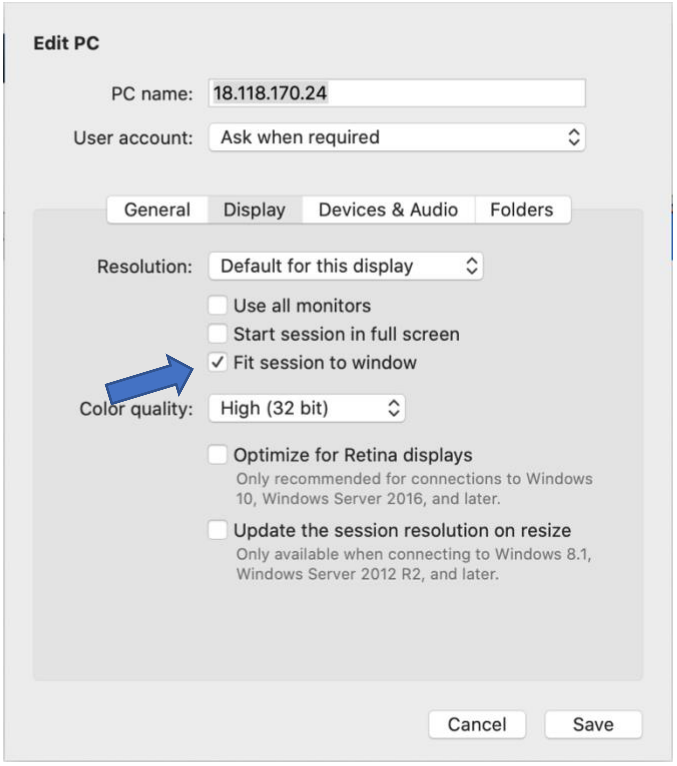

- Double click the card with the PC created

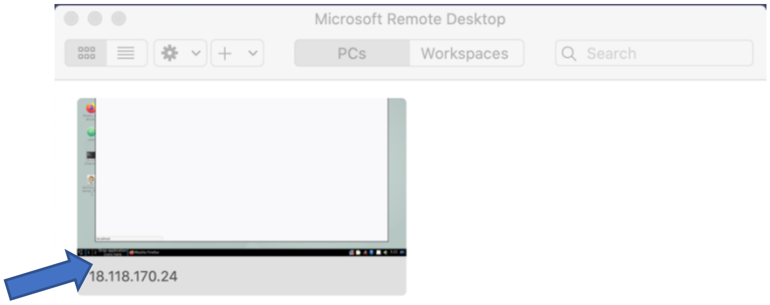

- Inform the **username = cds-user** and **password** you received for this workshop and click Continue

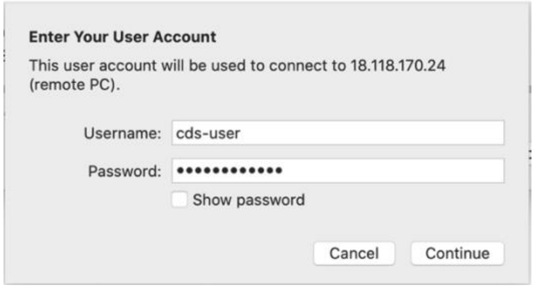

- After completing the login, the instance will be loaded. The Firefox Internet browser will open automatically to the Tutorial Home Page.

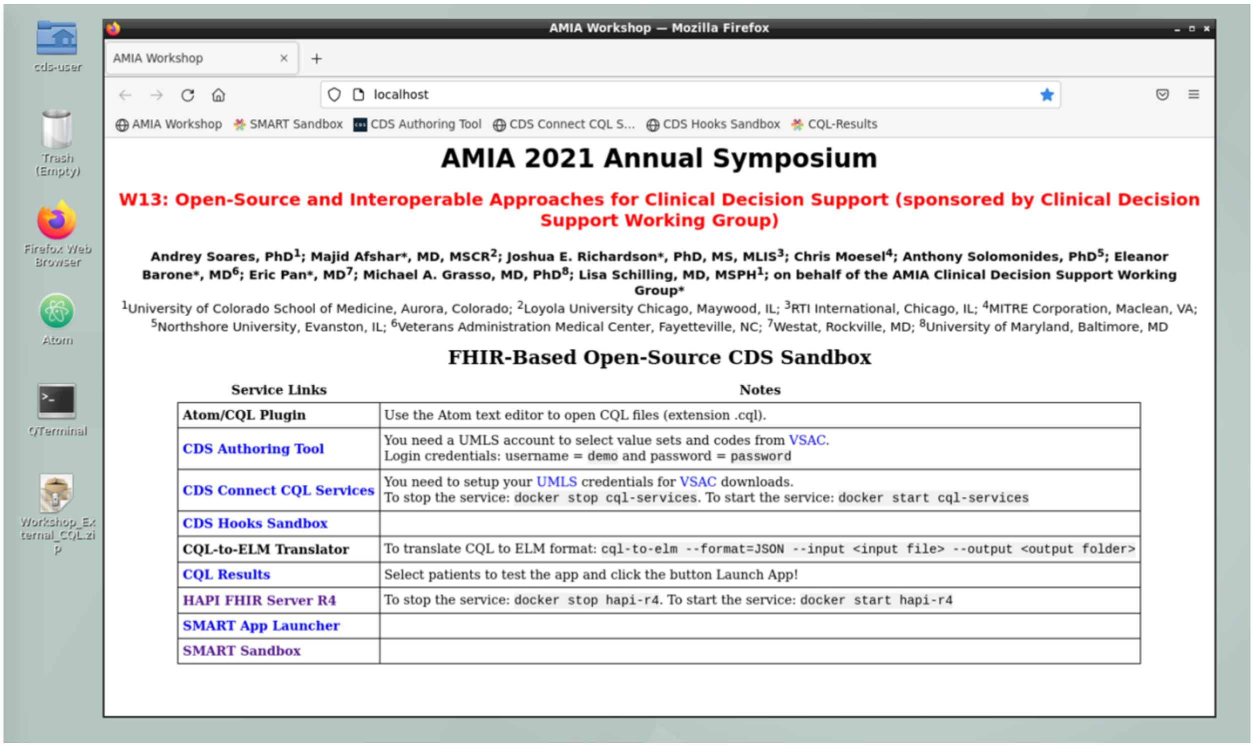

Setup UMLS Credentials

- Login and Password can be retrieved at http://uts.nlm.nih.gov/uts. In the left sidebar “UTS Profile”, select the option “Visit Your Profile”. You will be prompted to Sign In with your account.

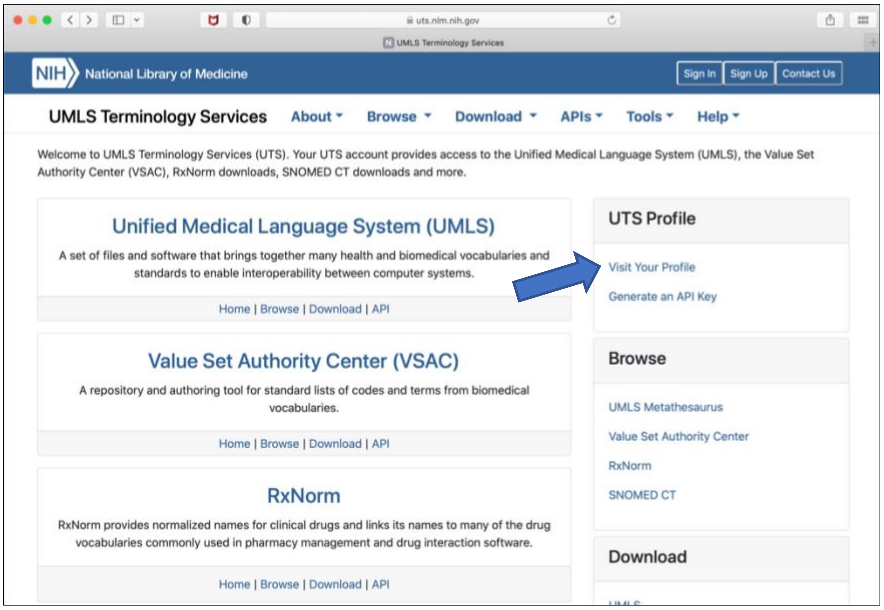

- For this workshop, you will need the **Username** and the **API KEY**.
- Open the QTerminal application (see QTerminal Icon on the Remote Desktop, not on your computer)
- Type:

```
nano .bashrc
```

- Scroll to the bottom of the page (using the arrow keys from your keyboard)
- Update the variables **UMLS_USER_NAME** and **UMLS_API_KEY** with your UMLS credentials

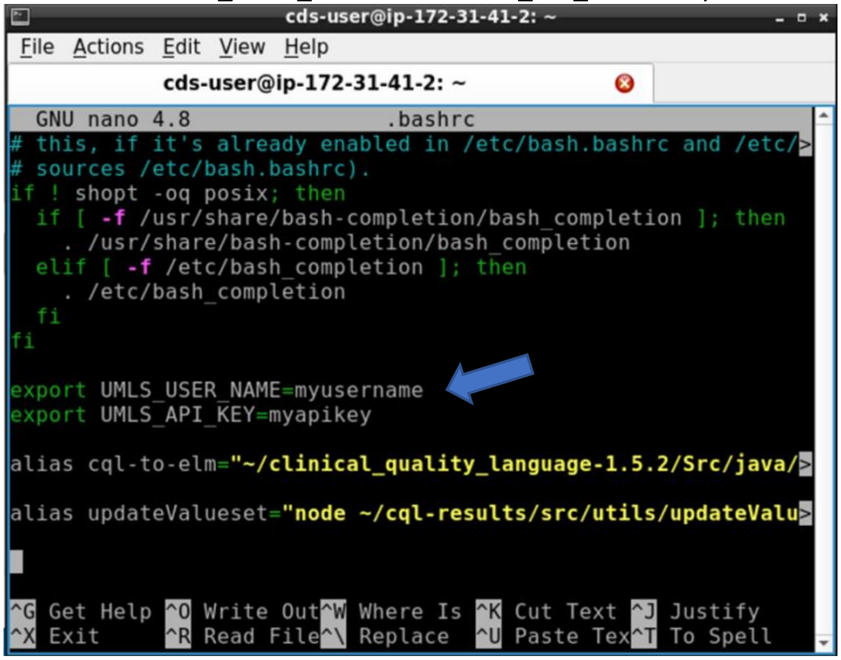

- Press **Ctrl + X** to exit. The editor will ask if you want to save the modified buffer. Press **Y**. Then the editor will ask to confirm the file name to write. Press **Enter**.
- In the terminal, type the following command to set the environment variables.
  Note: the command starts with a dot.

```
. ~/.bashrc
```

- To confirm the changes in the UMLS credentials, type the following command. It should show the variables with the correct information.

```
env | grep UMLS
```

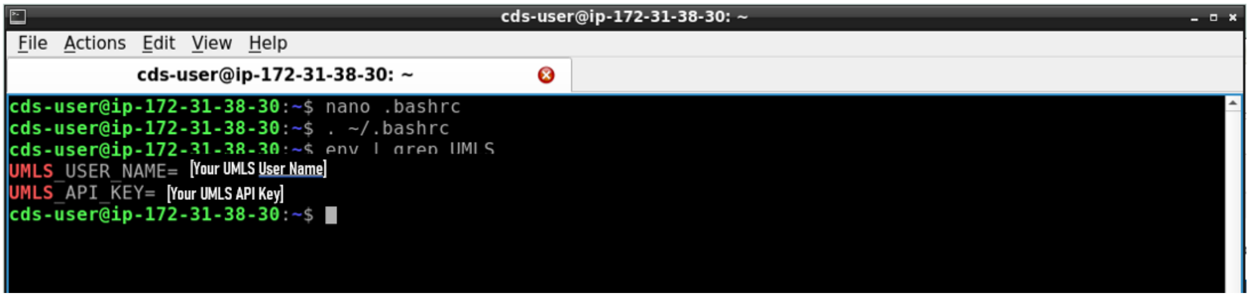

- Type the following command to setup the UMLS credentials to the CQL Services. It will stop the CQL services docker container and restart it with the new UML credentials that you setup above.

```
./scripts/setup_vsac.sh
```

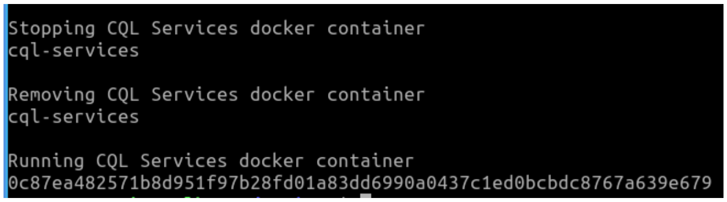

- Close the **QTerminal** window

Leaving the Instance

- If you need to leave the instance at any time, click the Icon on the bottom left, select the option **Leave** and then **Logout**.

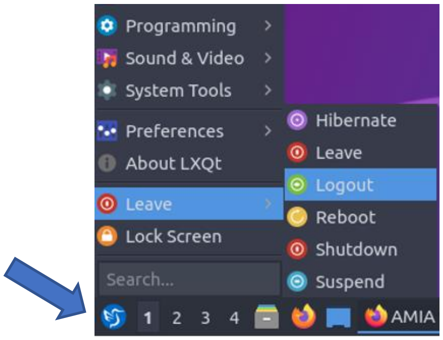

Hands-On Exercise Steps

YOU ARE HERE:

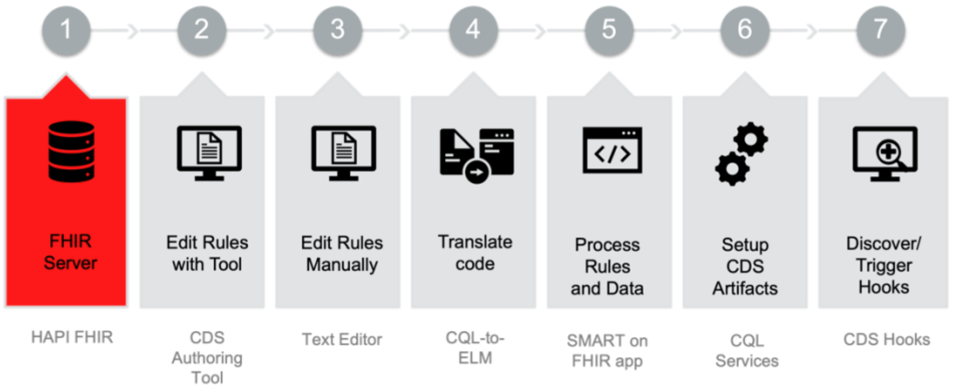

STEP 1: EXPLORE HAPI FHIR

Goal: To use the HAPI FHIR to search for appropriate patients to test the CDS logic. For this workshop, we also added new synthetic patient data to allow us to test them for statin recommendation.

The VM has a HAPI FHIR server with data from 644 synthetic patients, including over 100K observations, 4K conditions, 15K procedures and several other FHIR resources. The FHIR version used in this tutorial is FHIR R4 (version 4.0.1). FHIR STU3 and DSTU2 are disabled.

You may want to skip this step If…

- you are already familiar with the HAPI FHIR Server

This step is not a requirement to complete the other steps of this tutorial.
Go to **Step 2**.

1. To access the FHIR server, click the **SMART Sandbox** bookmark link below the URL bar and select the option **Open HAPI Server UI**. Alternatively, select the link HAPI FHIR Server R4 from the Tutorial Home Page

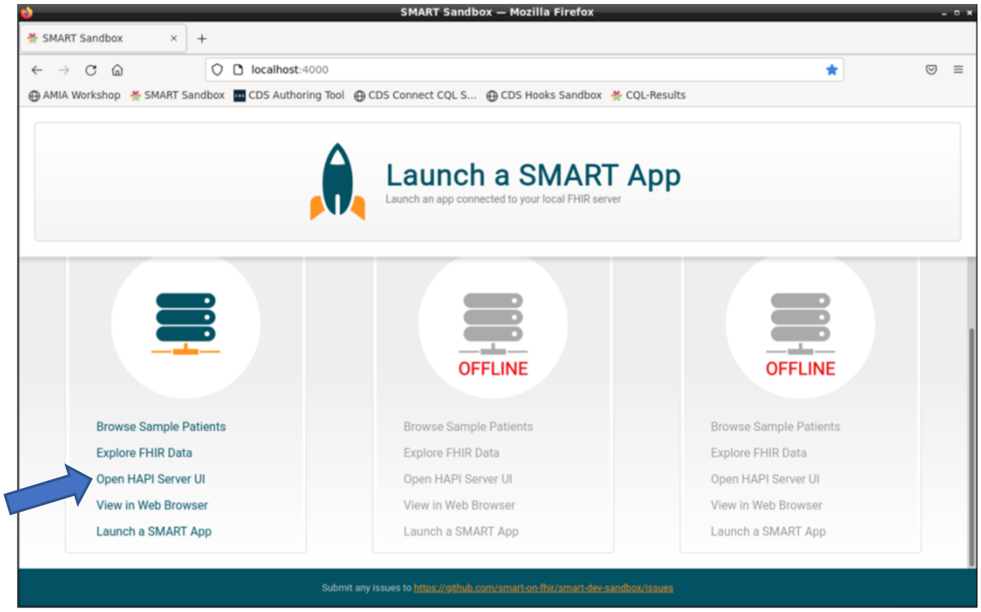

2. After selecting **Open HAPI Server UI**, HAPI FHIR will show a list of FHIR resources available for 644 patients.

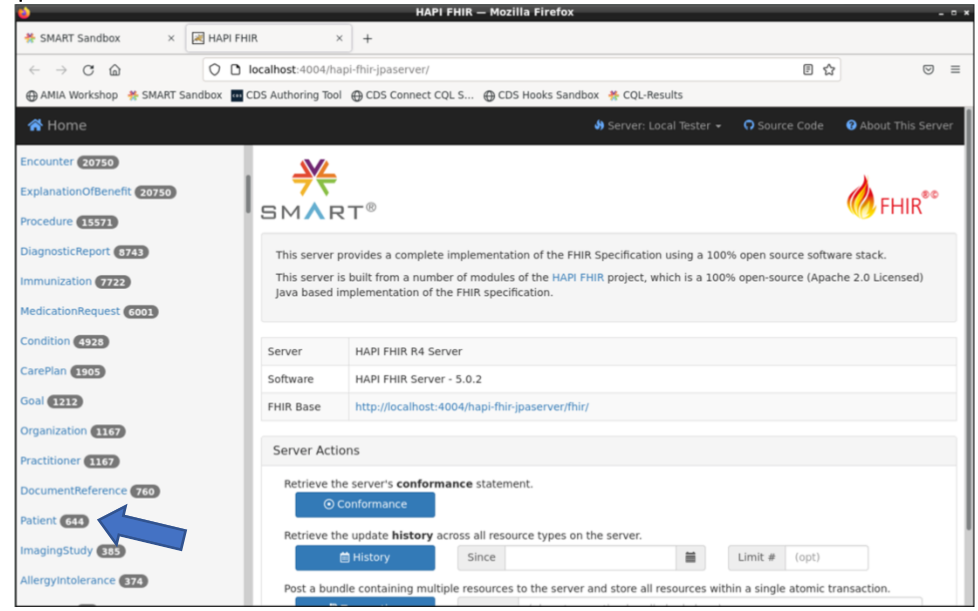

3. Return to the SMART Sandbox page (use the browser TAB)

4. The SMART Sandbox also contains a tool for browsing sample patients.
   1. Return to the SMART Sandbox bookmark link below the URL bar and select the option **Browse Sample Patients**.

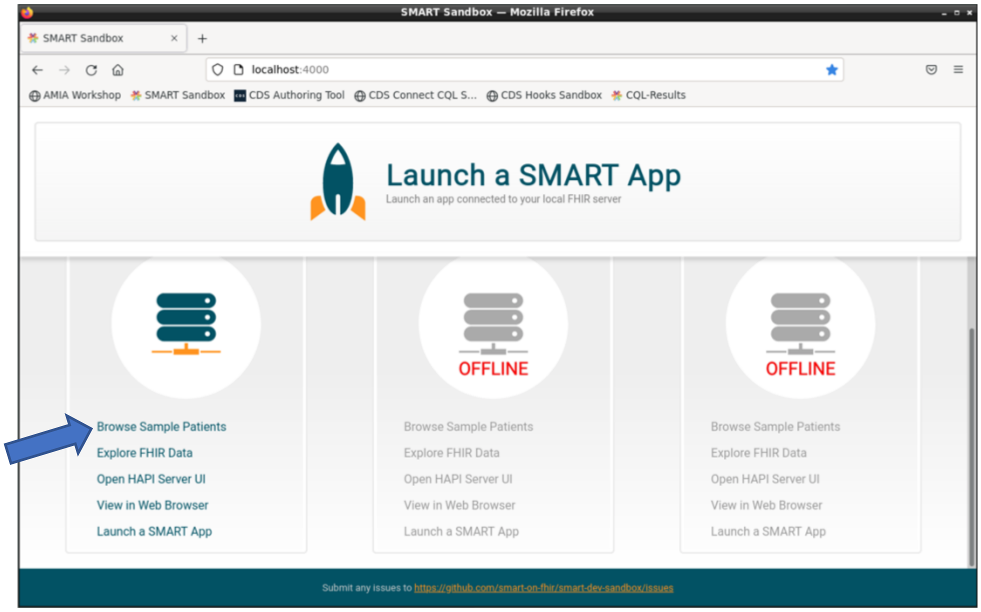

5. This tool allows you to search patients by name, gender, age, conditions, and tags. It also has an advanced search mode to allow search with a query string. For instance, we can search for patients with "Atopic Dermatitis".
    1. Select the **Conditions** tab
    2. Enter the term **Atopic Dermatitis** and select the condition from the dropdown list
    3. Click the **Search** button in the upper right.

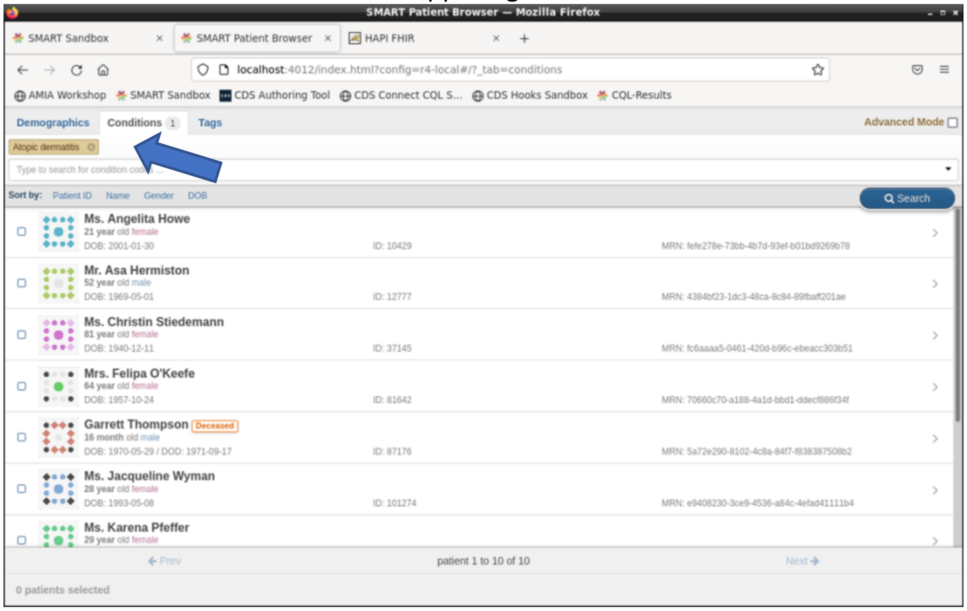

6. Click the first patient in the list **Ms. Angelita Howe** to view the list of FHIR resources available for this patient. The Resources will display in the left sidebar for you to see more details about Ms. Howe for whichever item you select. For example, the Observation - Laboratory Resources has the information seen in the screenshot below. Note that the gray jellybeans contain a number, which indicates the number of values currently available for that Resource. In this example, Ms. Howe has 52 laboratory results available as FHIR resources.

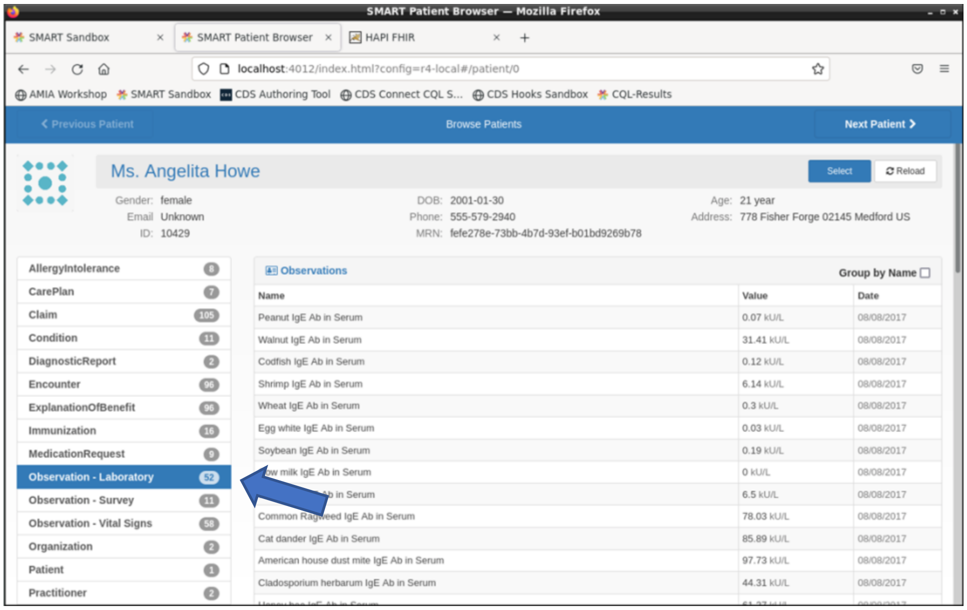
SMART Patient Browser — Mozilla Firefox
SMART Sandbox
SMART Patient Browser
HAPI FHIR
localhost:4012/index.html?config=r4-local#/patient/0
AMIA Workshop
SMART Sandbox
CDS Authoring Tool
CDS Connect CQL S...
CDS Hooks Sandbox
CQL-Results
Previous Patient
Browse Patients
Next Patient
Ms. Angelita Howe
Select
Reload
Gender: female
Email Unknown
ID: 10429
DOB: 2001-01-30
Phone: 555-579-2940
MRN: fefe278e-73bb-4b7d-93ef-b01bd9269b78
Age: 21 year
Address: 778 Fisher Forge 02145 Medford US
AllergyIntolerance 8
CarePlan 7
Claim 105
Condition 11
DiagnosticReport 2
Encounter 96
ExplanationOfBenefit 96
Immunization 16
MedicationRequest 9
Observation - Laboratory 52
Observation - Survey 11
Observation - Vital Signs 58
Organization 2
Patient 1
Practitioner 2
Observations
Group by Name
Name
Value
Date
Peanut IgE Ab in Serum 0.07 kU/L 08/08/2017
Walnut IgE Ab in Serum 31.41 kU/L 08/08/2017
Codfish IgE Ab in Serum 0.12 kU/L 08/08/2017
Shrimp IgE Ab in Serum 6.14 kU/L 08/08/2017
Wheat IgE Ab in Serum 0.3 kU/L 08/08/2017
Egg white IgE Ab in Serum 0.03 kU/L 08/08/2017
Soybean IgE Ab in Serum 0.19 kU/L 08/08/2017
0 kU/L 08/08/2017
6.5 kU/L 08/08/2017
78.03 kU/L 08/08/2017
Cat dander IgE Ab in Serum 85.89 kU/L 08/08/2017
American house dust mite IgE Ab in Serum 97.73 kU/L 08/08/2017
Cladosporium herbarum IgE Ab in Serum 44.31 kU/L 08/08/2017

YOU ARE HERE:

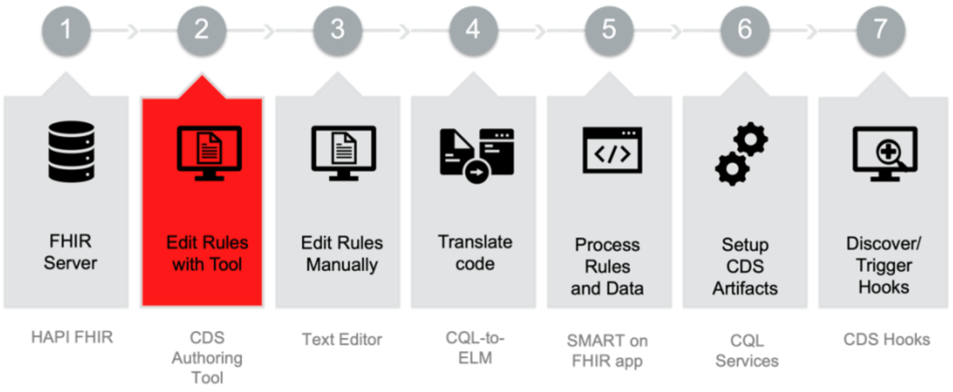

STEP 2: CREATING ARTIFACT WITH CDS AUTHORING TOOL

Goal: Use the CDS Authoring Tool to create CDS rules using CQL. A set of rules is referred to as an "artifact". The CDS Authoring Tools allows users to make use of other CQL artifacts and value set from the Value Set Authority Center (VSAC).

You may want to skip this step If…

- you don't have a UMLS account
- you are already familiar with the CDS Authoring Tool and just want to continue the tutorial

We have a rescue-files folder in the VM with the final CQL file resulting from this step.
Go to the **Step 3**.

2.1 Login to CDS Authoring Tool

1. Click the **CDS Authoring Tool** bookmark link below the URL bar on the Internet Browser
2. Click on the **Login** button (Top Right)

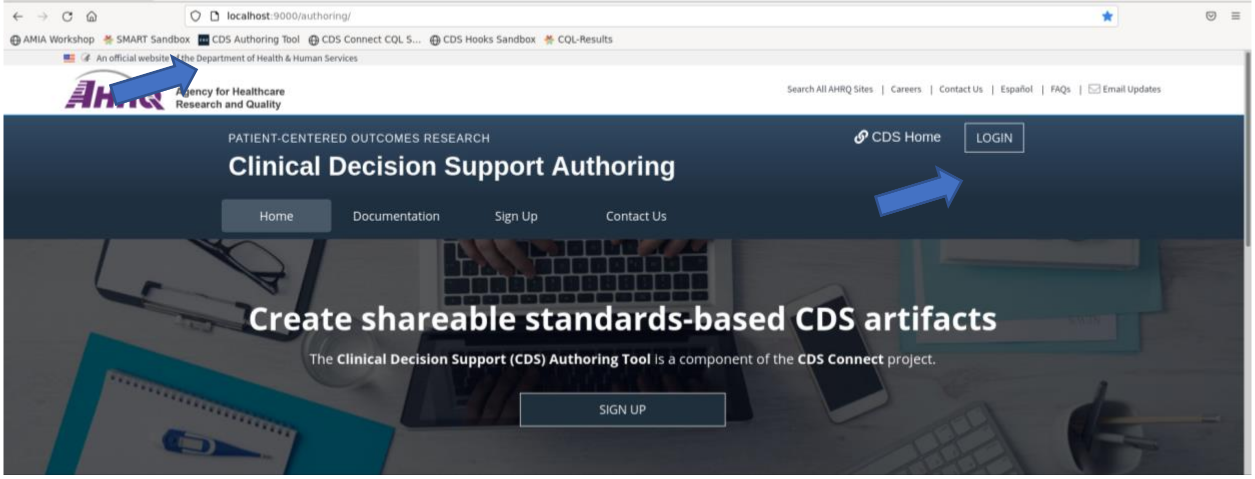

3. Login with the credential's username = **demo** and password = **password**. The browser will prompt you to save your password. Select Save or Don't Save, as you prefer.

2.2 Create the Statin Use Example Artifact

1. We will create an artifact to support statin prescribing based on a person's risk factors and ASCVD score. We're simplifying the CDS for the purpose of the tutorial.
    a. The 2016 USPSTF found adequate evidence that use of low- to moderate-dose statins reduces the probability of CVD events (MI or ischemic stroke) and mortality by at least a moderate amount in adults aged 40 to 75 years who have 1 or more CVD risk factors (dyslipidemia, diabetes, hypertension, or smoking) AND a calculated 10-year CVD event risk of 10% or greater.
    b. Our simple rule for purpose demonstration only is for a statin recommendation for persons ages 40-75 years, with (LDL >130 mg/dL and Has Diabetes) AND (ASCVD risk score > 10%).
2. If it is not already selected, click the **Artifacts** tab (Under the page title, "Clinical Decision Support Authoring")
3. Click the button labeled **"+ Create New Artifact"**

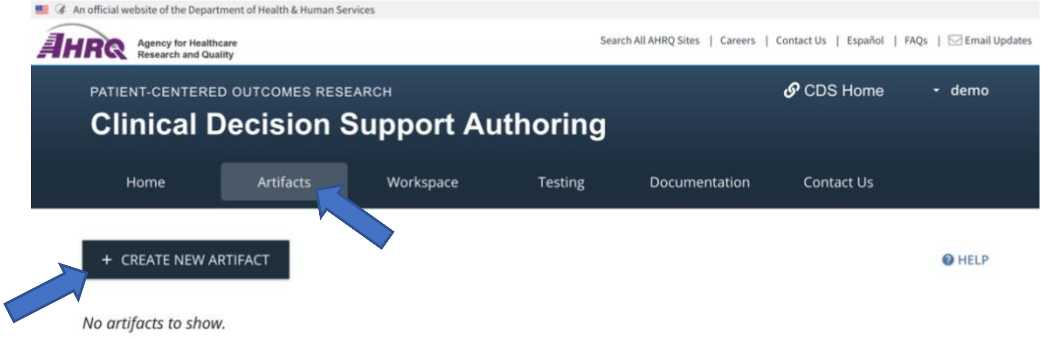

4. Enter the following information into the form and then click **"Create"**.
    a. Artifact Name: **Statin Use Example**
    b. Version: **0.0.1**

⚠ IMPORTANT: Double check the information (Name and Version) was entered correctly as they are used later in the workshop.
Note that the name has **first letters capitalized** and **spaces**.

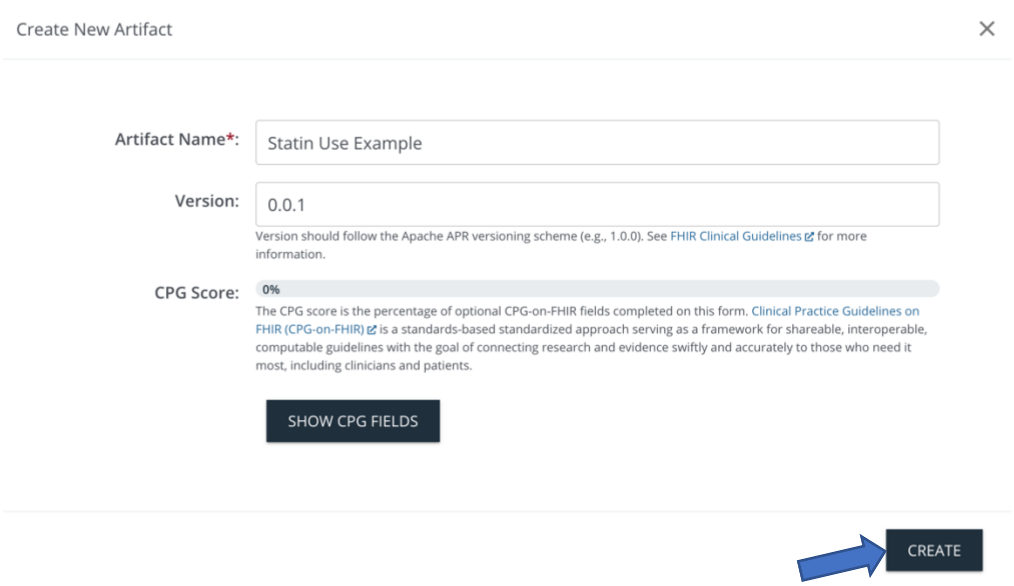

5. Click on the artifact name, **Statin Use Example**, in the artifact table

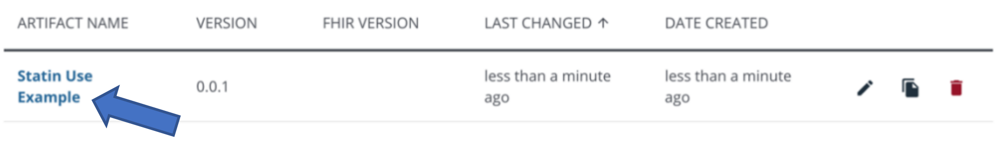

2.3 Import External CQL Libraries

To complete the Statin Recommendation CDS artifact we will need to calculate the ASCVD Risk of the patient. The risk score can be calculated using an external CQL artifact. The artifact "Million_Hearts_Baseline_10_Year_ASCVD_Risk_FHIRv400" can be imported and used inside our "Statin Use Example" artifact. The goal of this step is to demonstrate how to reuse existing CQL artifacts as part of new artifacts.

The Statin Use artifact requires two libraries:

- **Million_Hearts_Baseline_10_Year_ASCVD_Risk_FHIRv400**, which also requires the CDS_Connect_Commons_for_FHIRv400 and the CDS_Connect_Conversions
- **FHIRHelpers**, which is not imported because it is automatically generated by the CDS Authoring Tool.

To import external CQL libraries:

1. Select the **External CQL** tab

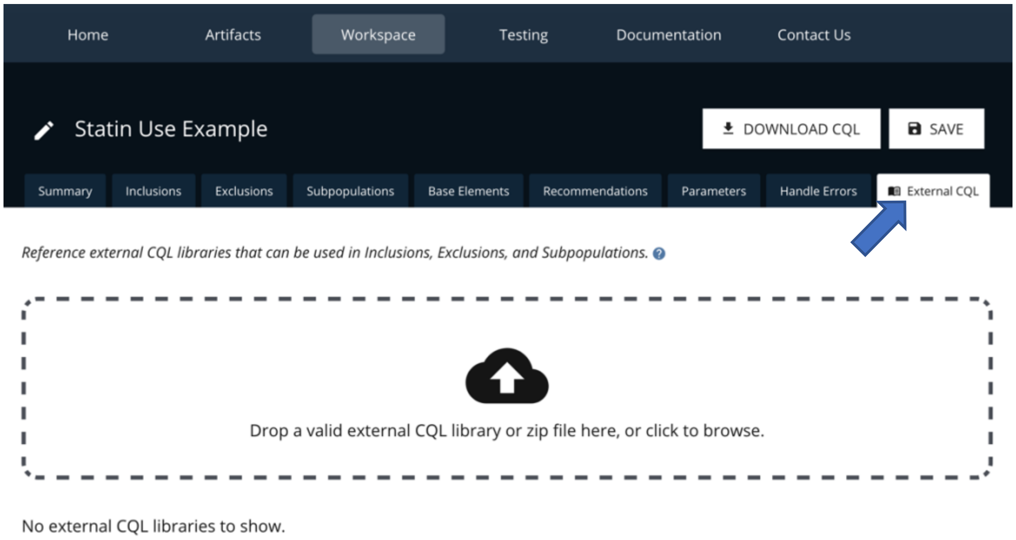

2. Click the cloud icon in the dashed area. It will open a window for **File Upload**.
3. Select **Desktop** on the left sidebar
4. Select the file **Workshop_External_CQL.zip** file (available on Desktop)
5. Click the button **Open** (Bottom Right)

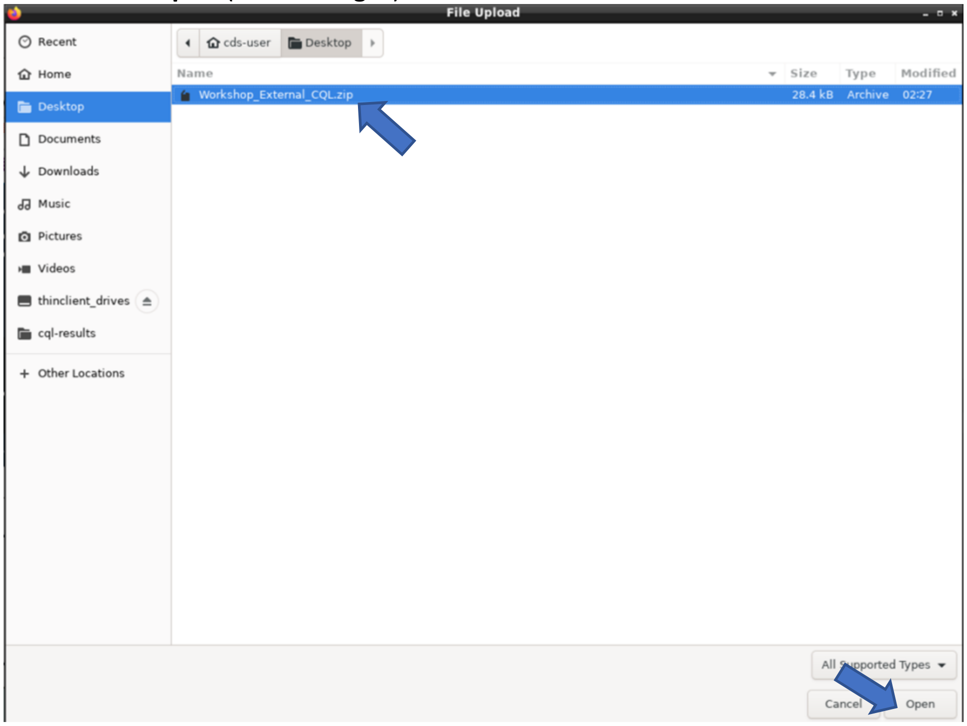

6. The system will import the files and display them as shown below. Scroll down to see them.

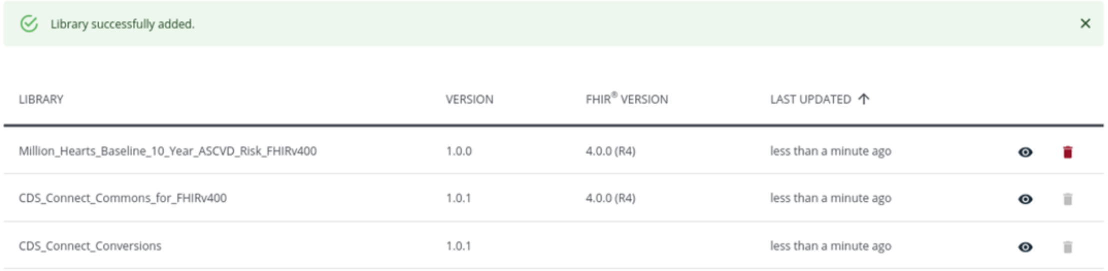

7. You have now successfully uploaded external CQL files that can be used in your artifact's logic.

2.4 Add Inclusion Criteria

1. Click the **Inclusions** tab

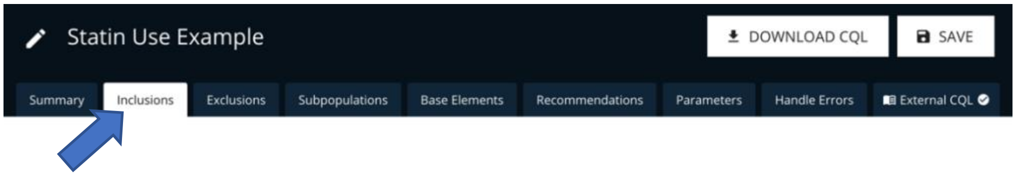

2.4.1 Age 40 to 75

First, we will enter inclusion criteria to specify that this statin CDS is only relevant to patients between the ages of 40 and 75 years.

1. Select "**Demographics**" from the "**New element**" menu
2. Select "Age Range" from the Demographics Element sub-menu
   a. Ignore for now the message: "You must specify Unit of Time and one of Minimum Age, Maximum Age."; this is expected and will be resolved as you continue to build the element
3. Enter the name of the element in the first input box of the form
   a. Age Range: **Age 40 to 75**

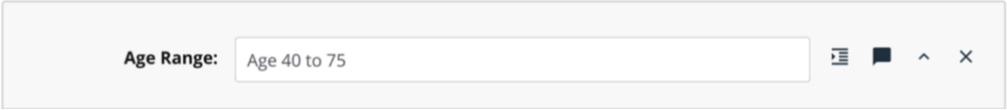

4. Enter the logical age range criteria in the form. The end point ages are included in the range.
   a. Minimum Age: **40**
   b. Maximum Age: **75**
   c. Unit of Time: **years**
   d. Return Type: **Boolean** (should already be selected)
   e. Once steps a-d are complete then this inclusion criteria is now complete

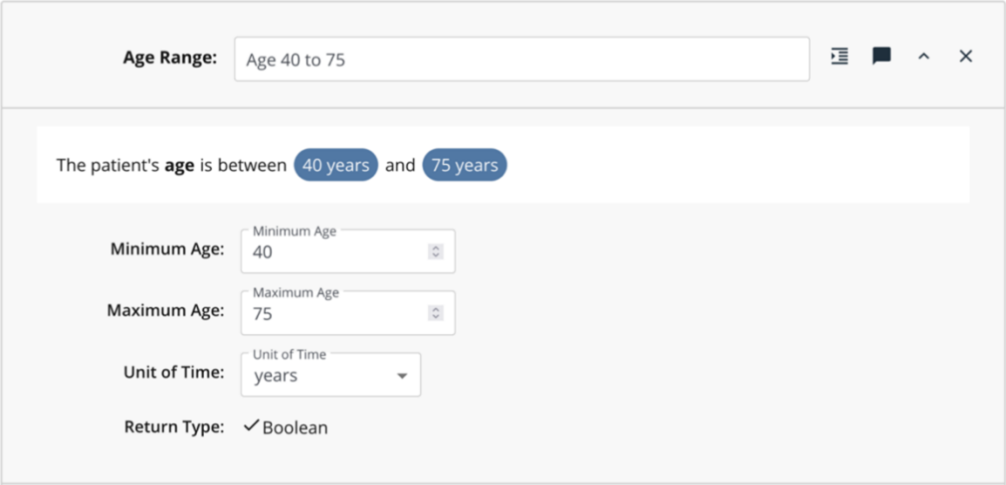

2.4.2 LDL > 130 mg/dL

Next, we want to specify the inclusion criteria: LDL > 130 mg/dL. You should see the "AND" operator selected below the element you just created. We will create the next rule for LDL below that. For this CDS, we've determined that we're only interested in the **most recent** and **verified** result with a value **LDL > 130 mg/dL**, as long as it is within the past (**Look Back**) 6 years.

1. Scroll down to the "**New element**" menu in the next element on the page and select "**Observation**"

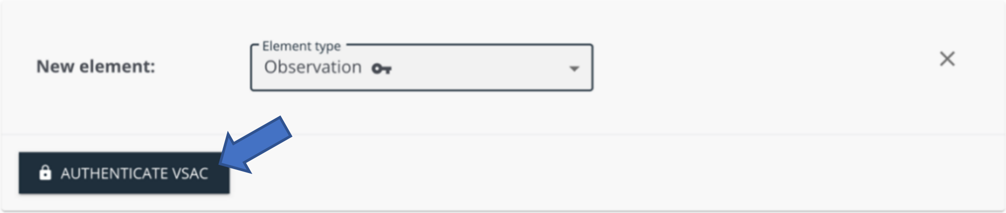

2. Click "**Authenticate VSAC**" and log into the VSAC using your UMLS API key.
3. Enter your UMLS Terminology Services **API key**, then click "**Login**". If the browser prompts you to save your password, select Save or Don't Save, as you prefer.

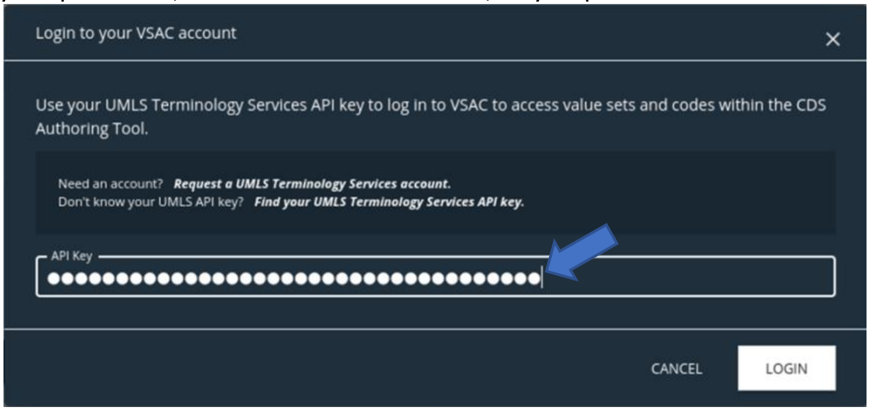

4. You should be brought back to the "New Element" menu after login.
5. Click the "**Add Value Set**" button.

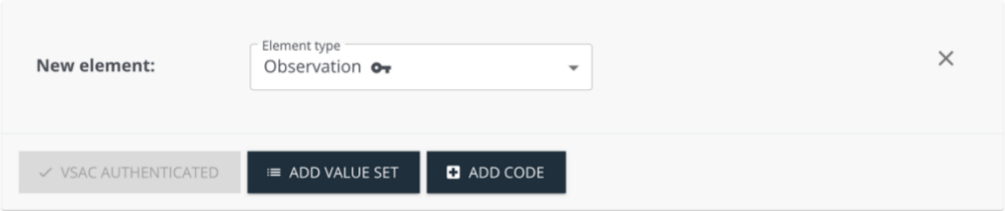

6. Enter "**LDL-c**" and click "**Search**"
    a. *Note: the "LDL-c" is not case sensitive*
7. Select "LDL-c" value set with OID "2.16.840.1.113883.3.117.1.7.1.215"
    a. *Note: The Steward is "TJC EH Steward"*
    b. Ignore for now the message: ""Element must have return type 'boolean'"; this will be resolved as you continue to build the element
8. Enter a new name for the element in the input box of the form
    a. Observation: **LDL > 130 mg/dL**

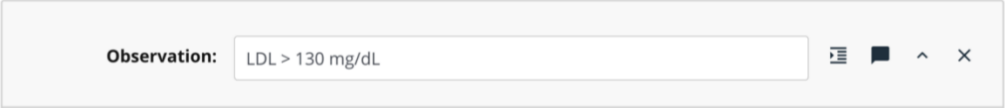

9. Click "**Add Modifiers**" to further refine the criteria
    a. Choose **"Select Modifiers"** to use a built-in expression modifier
10. Select **"Look Back"** from the modifier menu and enter the following:
    a. Value: **6**
    b. Unit: **Year(s)**
11. Select the modifier "**Verified"**
12. Select the modifier "**Most Recent**"
13. Select the modifier "**Quantity Value**"
    a. *DO NOT choose the "QuantityValue" expression from the Million Hearts library*
14. Select the modifier **Value Comparison** and enter the following:
    a. minOp: **>**
    b. minValue: **130**
    c. maxOp and maxValue: **leave blank**
    d. unit: **mg/dL**
15. Click **"Add"** to add the modifier and return to the workspace

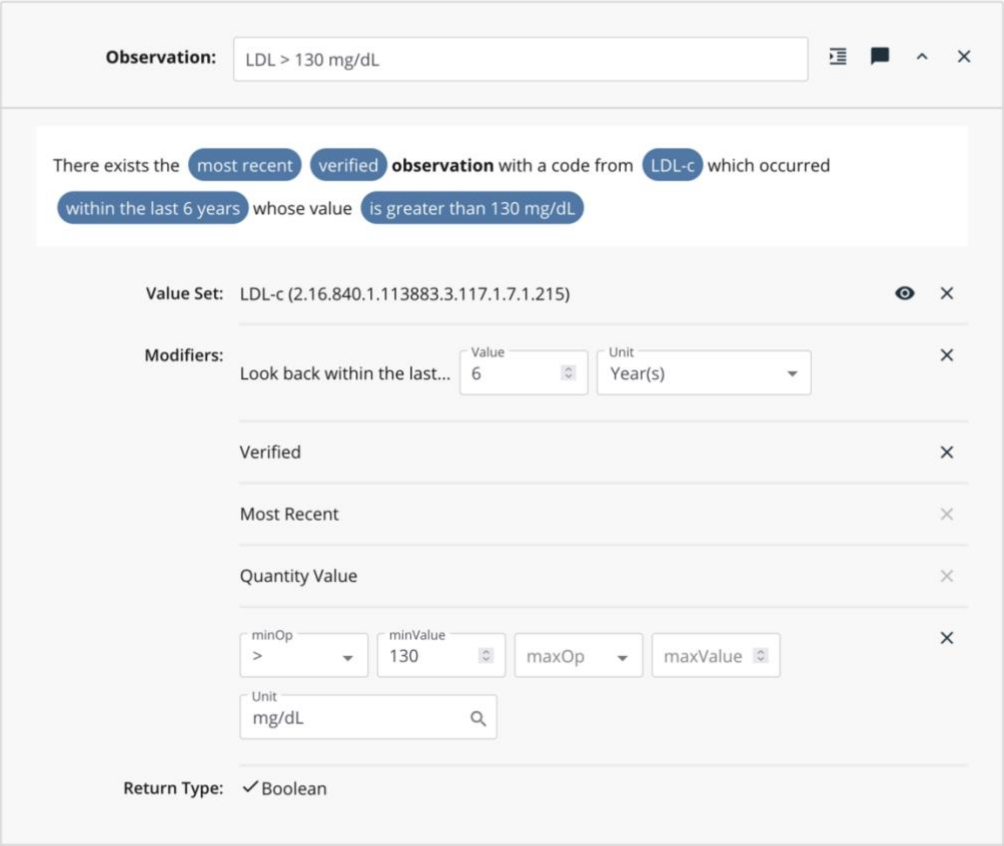

2.4.3 Create Group

This Group will maintain the possible risk factors that are key to computing the ASCVD risk and informing the Statin Recommendation. Although the original CDS specified several risk factors, we will only add one more risk factor for this exercise. We group the risk factors together, using the “OR” conjunction, since the patient only needs evidence of one risk factor to qualify for this portion of the inclusion criteria.

1. In the **LDL > 130 mg/dL** expression created above, click the button 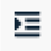 (On the right) to indent and create a Group of criteria
2. Enter the name of the group in the input box of the form
    a. Group: **One or More Risk Factors**

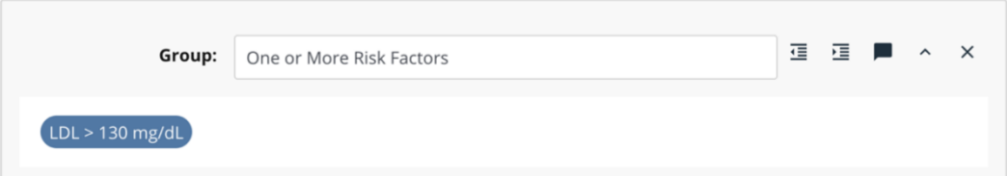

2.4.4 Has Diabetes

In addition to LDL > 130 mg/dL, the other risk factor we will look for is presence of Diabetes. For this example, we will use the brand new “Build New Modifier” feature to filter Diabetes records to only those that are **confirmed** and **active**.

1. Inside the Group, select "**Condition**" from the "**New element**" menu in the next element on the group (directly below the **"Or"** operator)
2. Click the "**Add Value Set**" button
3. Enter "**Diabetes**" and click the button "**Search**"
4. Select "Diabetes" value set with OID "2.16.840.1.113883.3.464.1003.103.12.1001"
    a. *Note: The Steward is "NCQA PHEMUR"*
5. Enter the name of the element in the input box of the form
    a. Condition: **Has Diabetes**

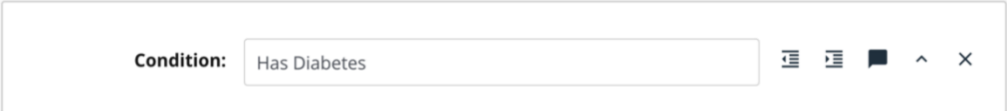

6. Click "**Add Modifiers**" on the Diabetes element to further refine the criteria
7. Click **"Build New Modifier"** to define filter criteria using properties of Condition
8. Under the top-level **"AND"** group, click **"Add Rule"** and select the following:
    a. Property: **Verification Status**
    b. Operator: **Matches Standard Code in**
    c. Code(s): **confirmed**
9. Below the rule you just defined, click **"Add Rule"** again and select the following:
    a. Property: **Clinical Status**
    b. Operator: **Matches Standard Code in**
    c. Code(s): **active**

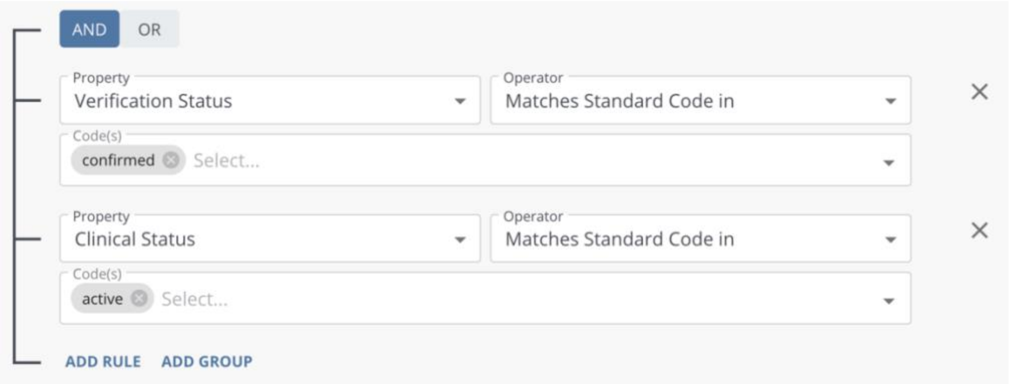

10. Click the **"Add"** button (lower right corner) to add the new modifier criteria
11. Click **"Add Modifiers"** and then **"Select Modifiers"** to further refine the criteria
12. Select the modifier **"Exists"** to indicate that this element must be in the patient's record
13. Click **"Add"** to add the modifier and return to the workspace

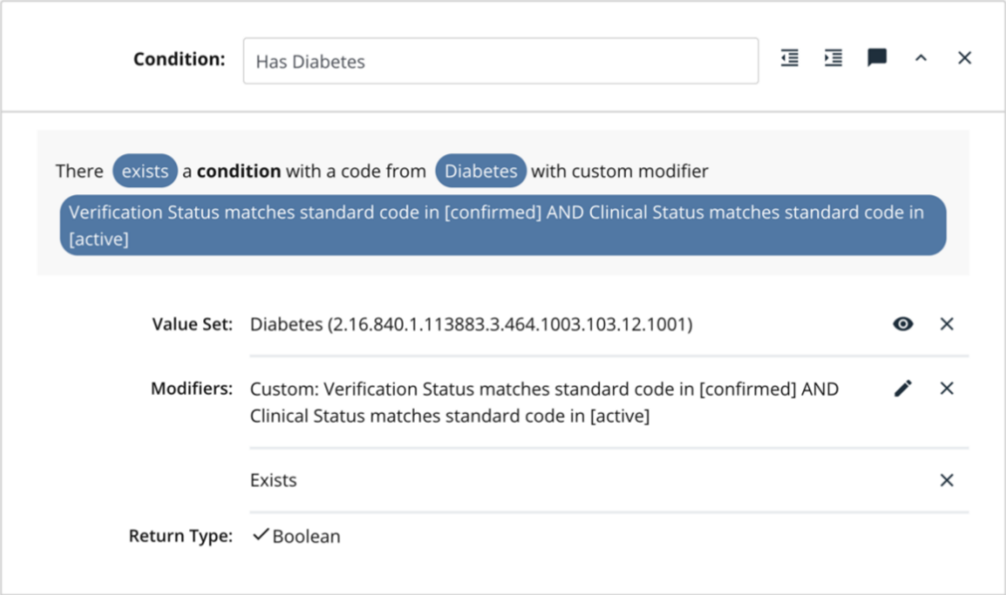

2.4.5 10-year ASCVD Risk >= 10%

The last inclusion criteria we will specify in this exercise is that the patient must also have a 10-year ASCVD risk score of at least 10%. This is the threshold required for the Grade B recommendation for a statin. Although the original CDS also covers the Grade C recommendation (when 10-year ASCVD risk is at least 7.5% but less than 10%), we will not cover that in this exercise.

The CDS Authoring Tool does not provide capabilities for authoring complex mathematical algorithms, so we will use a CQL file that was authored outside of the tool in order to use its 10-year ASCVD risk calculation logic. You already uploaded this external CQL in a previous step (2.3). Note that this external CQL library returns the risk as a decimal, so 10% risk is indicated by the decimal score 0.1.

1. **Outside** of the Group, select "**External CQL**" from the "**New element**" menu in the last element on the page (directly below the last **"And"** operator)
2. Select the external library “Million_Hearts_Baseline_10_Year_ASCVD_Risk_FHIRv400”
3. Select definition “**PatientBaselineRisk**” (you may begin typing the name to more easily find it in the list)
4. Replace the name of the element in the input box of the form
    a. External CQL Element: **10-year ASCVD Risk >= 10%**

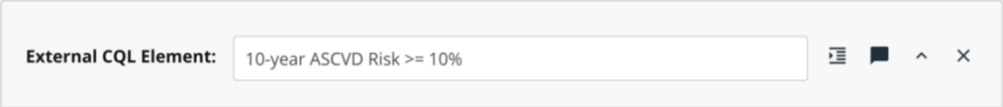

5. Click "**Add Modifiers**" and then **“Select Modifiers”** to further refine the criteria
6. Add the modifier **Value Comparison** and enter the following:
    a. minOp: **>=**
    b. minValue: **0.1**
    c. maxOP and maxValue: **leave blank**
7. Click **“Add”** to add the modifier and return to the workspace

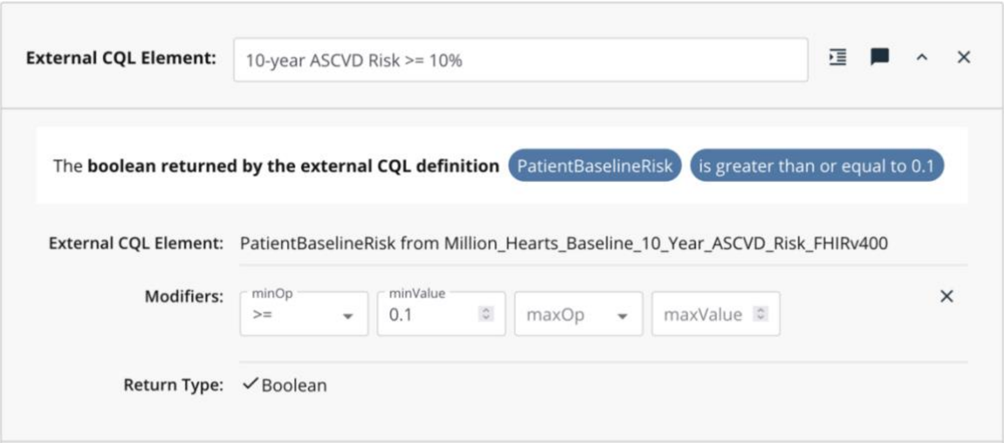

8. Click the button **Save** on the top right of the page to save changes in your artifact

2.5 Add Exclusion Criteria

1. Click the **Exclusions** tab.

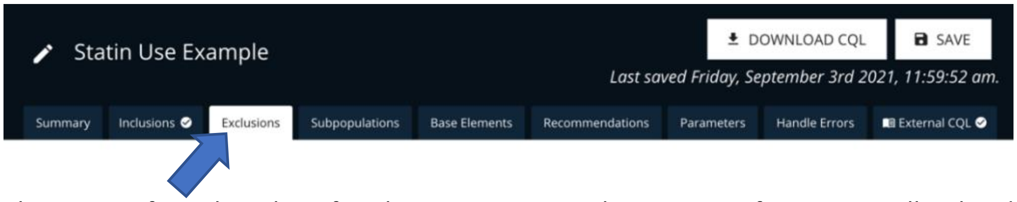

The original CDS specifies a long list of exclusion criteria. In the interest of time, we will only add one exclusion, indicating that the patient shouldn't receive a recommendation if they are already on statin therapy.

2.5.1 On Statin Therapy (FHIR MedicationRequest Resource)

If a patient is already on statin therapy, there is no need to recommend they consider statin therapy. In this case, we want them excluded. We will look for active or completed medication requests (e.g., prescriptions) from the last two years. The original CDS also looks for medication statements, but this exercise will focus only on requests. The FHIR MedicationRequest resource represents the medication ordered or requested by a provider for the patient.

1. Select "**MedicationRequest**" from the "**New element**" menu in the **Exclusions** tab
2. Click the "**Add Value Set**" button
3. Enter "**Statins**" and clicks "**Search**"
4. Select "Statins" value set with OID " 2.16.840.1.113883.3.3616.200.110.102.2050"
    a. *Note: The Steward is "Clinical Architecture"*
    b. *Note: The original CDS specifies six different value sets to use when querying for statins. In the interest of time and simplicity, we have chosen the largest statins value set in VSAC for this exercise instead.*
5. Enter the name of the element in the input box of the form
    a. Medication Request: **On Statin Therapy**

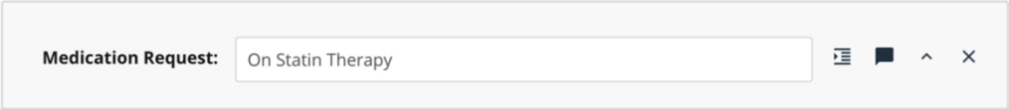

6. Click "**Add Modifiers**" on the Diabetes element to further refine the criteria
7. Click **"Build New Modifier"** to define filter criteria using properties of MedicationRequest
8. Under the top-level **"AND"** group, click **"Add Rule"** and select the following:
    a. Property: **Status**
    b. Operator: **Matches Standard Code in**
    c. Code(s): **active**, **completed** (select both independently)
9. Below the rule you just defined, click **"Add Rule"** again and select the following:
    a. Property: **Authored On**
    b. Operator: **Occurred Within Last**
    c. Value: **2**
    d. Time Unit: **year(s)**

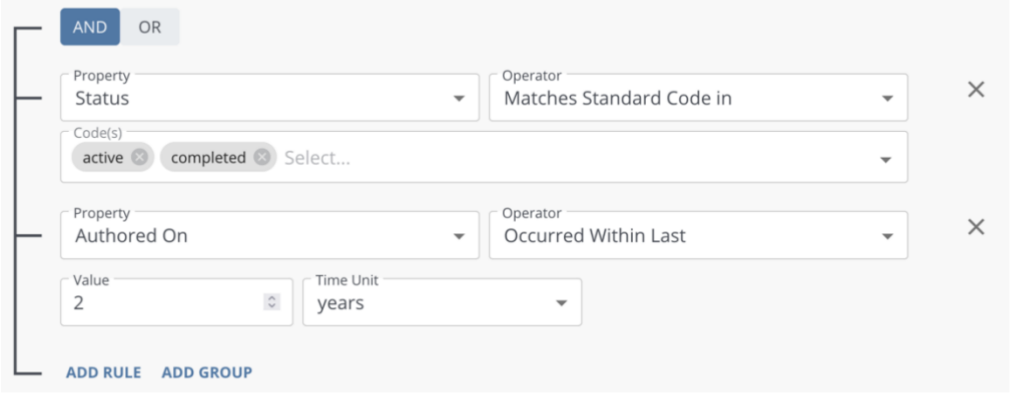

10. Click **"Add"** to add the new modifier criteria
11. Click **"Add Modifiers"** and then **"Select Modifiers"** to further refine the criteria
12. Select the modifier **"Exists"** to indicate that this element must be in the patient's record
13. Click **"Add"** to add the modifier and return to the workspace

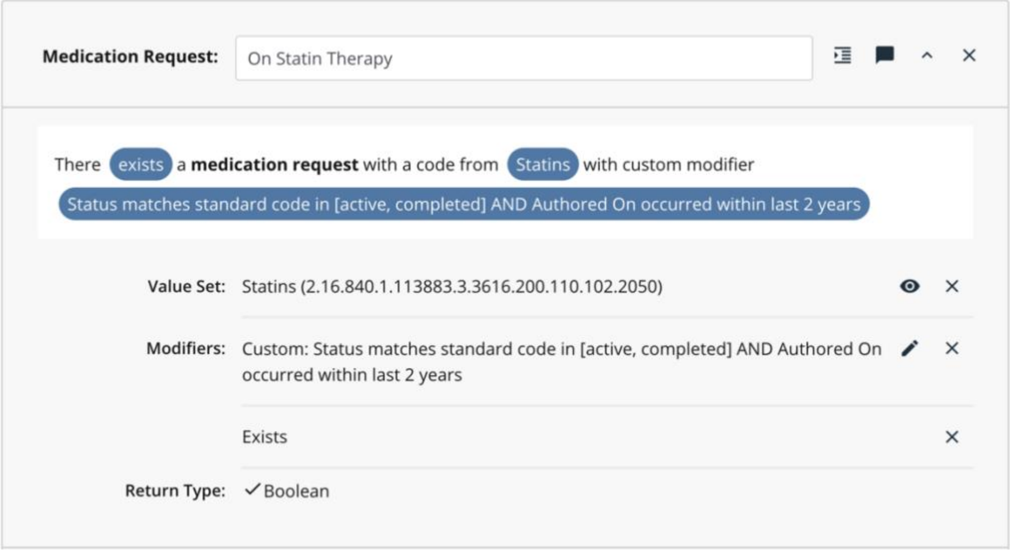

14. Click the button **Save** on the top right of the page to save changes in your artifact

2.6 Add Recommendations

- Scroll back to the top of the CDS Authoring Tool. Click the **Recommendations** tab.

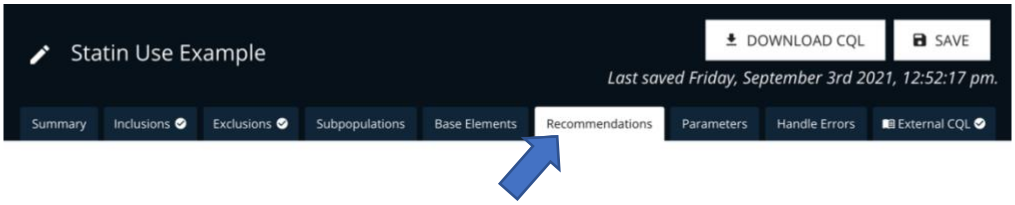

Although the original artifact could provide either a Grade B or Grade C recommendation, we have modified this example to apply only to the Grade B recommendation (when 10-year ASCVD risk is at least 10%).

1. Click the button **New Recommendation**
2. Enter the following recommendation (Copy/Paste):
   Start low to moderate intensity lipid lowering therapy based on outcome of shared decision making between patient and provider.
3. Click the button **Add Rationale** and enter (Copy/Paste):
   The USPSTF found adequate evidence that use of low- to moderate-dose statins reduces the probability of CVD events (MI or ischemic stroke) and mortality by at least a moderate amount in adults aged 40 to 75 years who have 1 or more CVD risk factors (dyslipidemia, diabetes, hypertension, or smoking) and a calculated 10-year CVD event risk of 10% or greater.

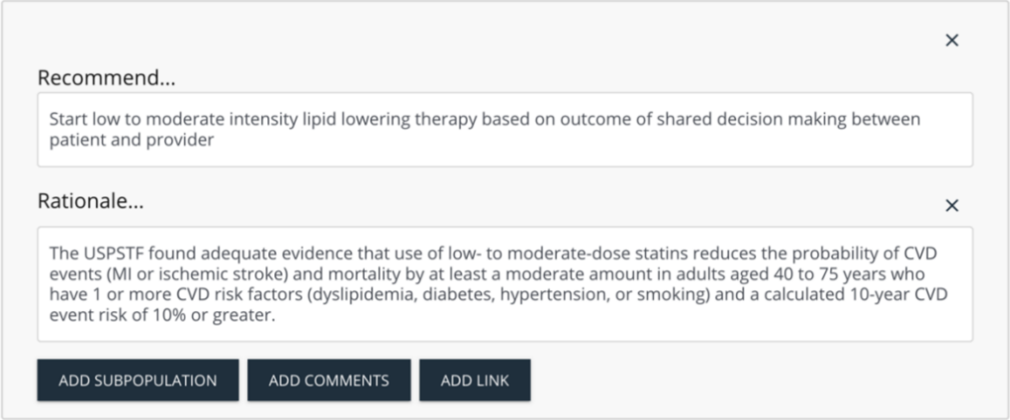

2.7 Save and Download CQL

1. Scroll back to the top of the CDS Authoring Tool. Click the button **Save** on the top right of the page to save changes in your artifact
2. Click the button **DOWNLOAD CQL** and select the option for **FHIR R4**

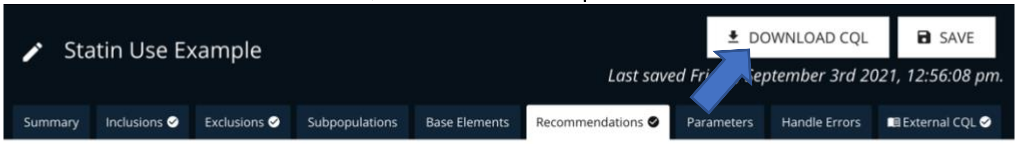

3. Choose the **"Save File"** option and click the button **OK** to save the file to the folder **Downloads**

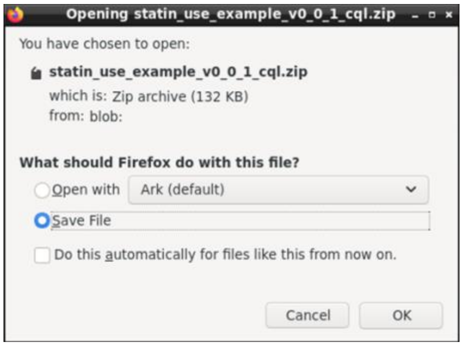

2.8 Review the new artifact

1. In File Manager (“cds-user” icon on Desktop), open the folder **Downloads**

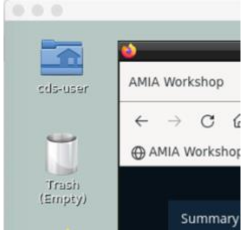

2. Navigate to the **Downloads** folder to find the file (**statin_use_example_v0_0_1_cql.zip**) downloaded from the CDS Authoring Tool
3. Double click the file and select the option **Extract** from the Menu Bar.

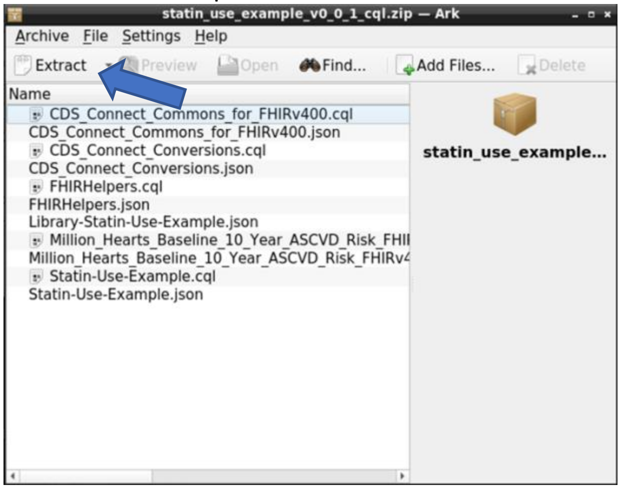

4. Click the button **Extract (**bottom of the window). It will extract the content of the zip file to the folder **statin_use_example_v0_0_1_cql** in the **Downloads**

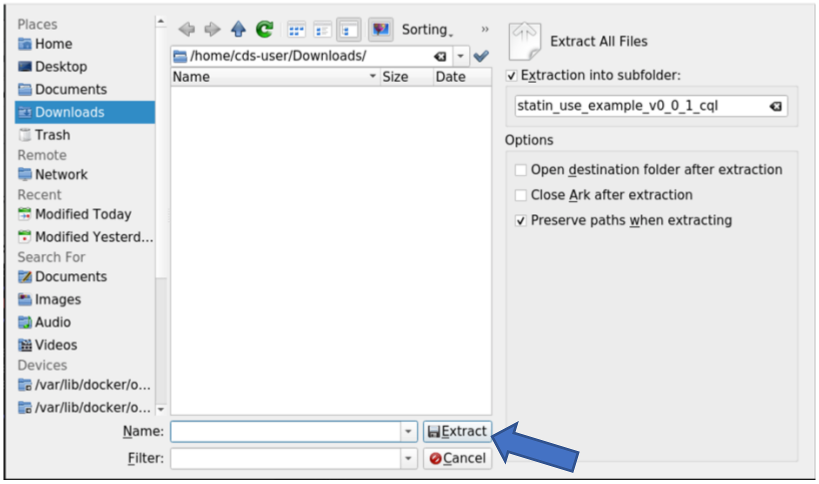

5. Close the ARK window (extraction tool)
6. In File Manager, open the folder **statin_use_example_v0_0_1_cql**
7. Double click the file **Statin-Use-Example.cql** to open it with the default text editor (FeatherPad)
8. Review the code generated by the CDS Authoring Tool (e.g., included libraries, value sets and codes, inclusion criteria, exclusion criteria, subpopulation, and recommendations).
9. Close the text editor
10. Close the File Manager window
11. In the Internet Browser, log out of the CDS Authoring Tool by clicking the username **demo** and choosing “Logout”

YOU ARE HERE:

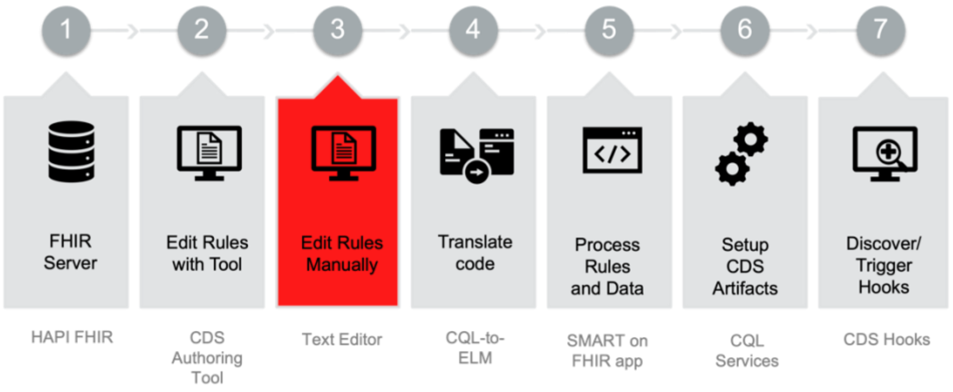

STEP 3: MANUALLY EDITING THE ARTIFACT

GOAL: In this step we will modify the CQL file generated by the CDS Authoring Tool to include a new inclusion criterion "Has Hypertension". The goal of this step is to show the process of building CQL artifacts manually using a text editor, which would be an option to write CQL expressions not yet implemented on the CDS Authoring Tool. In the previous step, we imported a CQL file to calculate 10-year ASCVD risk that was created manually because the CDS Authoring Tool does not provide capabilities for authoring complex mathematical algorithms.

You may want to skip this step If…

- you are not interested in writing code manually and you just want to continue the tutorial
- you are behind and want to catch up

We have a rescue-files folder in the VM with the final CQL file resulting from this step. Go to **Step 4**.

3.1 Copy the Statin-Use-Example artifact CQL to the CQL Results app

If you didn't skip the previous **Step 2**:

1. Open the **QTerminal** (see icon in the Desktop) and type `step3.1` to copy the files Statin-Use-Example (CQL and JSON) and Million_Hearts_Baseline_10_Year_ASCVD_Risk_FHIRv400 (CQL and JSON), downloaded (and extracted) from the CDS Authoring Tool to the folder cql-results/src/data/R4
2. Close the **QTerminal**

If you skipped the previous **Step 2**:

1. Open the **QTerminal** and type `skip2` to copy the files Statin-Use-Example (CQL and JSON) and Million_Hearts_Baseline_10_Year_ASCVD_Risk_FHIRv400 (CQL and JSON) from the folder rescue-files to the folder cql-results/src/data/R4
2. Close the **QTerminal**

3.2 Edit the Statin-Use artifact

1. In File Manager ("cds-user" icon on Desktop), Navigate to the folder **cql-results/src/data/R4**
2. Double click the file **Statin-Use-Example.cql** to open it in the text editor
3. Follow the instructions below to add a new inclusion criteria to the Statin-Use artifact

3.2.1 Add Alias to Library

1. Add the expression **`called C3F`** after the include "CDS_Connect_Commons_for_FHIRv400" version '1.0.1'

```
include "CDS_Connect_Commons_for_FHIRv400" version '1.0.1' called C3F
```

3.3 New inclusion Criteria: Has Hypertension

3.3.1 Add value sets

1. Add the following code to include new value sets to help identify Hypertension. Copy and paste the value set to below `valueset "Statin VS"`
   a. *Note: It is important to put them in the right place because CQL defines a specific order of item declarations*
   b. If you have trouble copy/pasting, try right-clicking and using the context menu to copy/paste between your system and the virtual machine

```
valueset "Hypertension VS":
'https://cts.nlm.nih.gov/fhir/ValueSet/2.16.840.1.113762.1.4.1032.9'
```

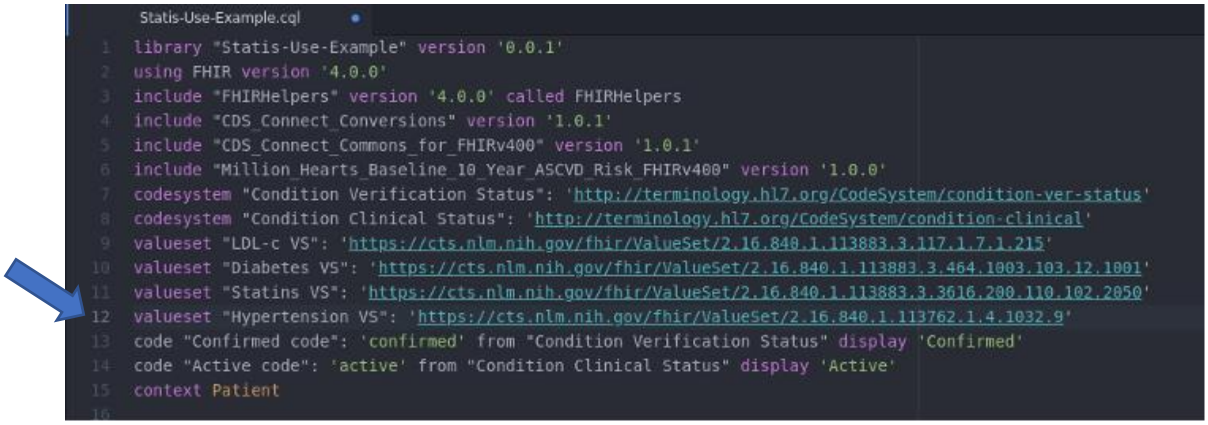

3.3.2 Define "Has Hypertension"

1. Add this code below the line with "`context Patient`". This code checks if there exists an active or recurring confirmed condition of Hypertension.
   Note: Leave a blank line before and after

```
define "Has Hypertension":
  exists(
     C3F.ActiveOrRecurring(
        C3F.Confirmed(
           [Condition: "Hypertension VS"]
        )
     )
  )
```

The result “Has Hypertension” definition should look like this.

```
define "Has Hypertension":
  exists(
    C3F.ActiveOrRecurring(
      C3F.Confirmed([Condition: "Hypertension VS"]
      )
    )
  )
```

3.3.3 Add “Has Hypertension” to the list of exclusion criteria

1. Add the following code to the end of the "One or More Risk Factors" definition.

```
or "Has Hypertension"
```

Here is an example of the final code for the "One or More Risk Factors" definition.

```
define "One or More Risk Factors":
  "LDL > 130 mg/dL"
  or "Has Diabetes"
  or "Has Hypertension"
```

3.4 Save the Statin-Use-Example artifact

1. Save your changes (File -> Save)
2. Close the text editor
3. Close the File Manager

YOU ARE HERE:

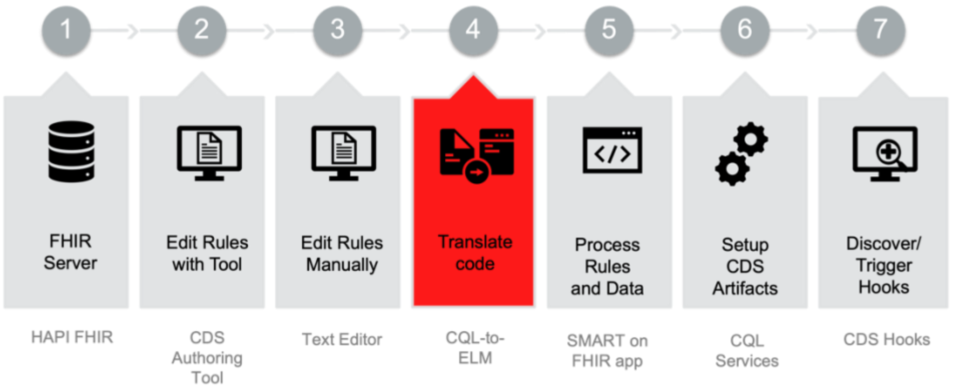

STEP 4: TRANSLATE CQL TO ELM

GOAL: To demonstrate how to manually translate the CQL file into JSON (ELM). After modifying the source CQL artifact, we need to translate it to JSON so that it can be used by a CQL Engine. The translated fille will be use in other steps of this tutorial.

You may want to skip this step If…

- you are not interested in writing code manually and you just want to continue the tutorial
- you are behind and want to catch up

We have a rescue-files folder in the VM with the final CQL file resulting from this step. Go to **Step 5**.

If you skipped the previous **Step 3**:

1. Open the **QTerminal** and type `skip3` to copy the file **Statin-Use-Example.cql** file to the folder **cql-results/src/data/R4**
2. Close the **QTerminal**

4.1 Translate Artifact

1. Open the **QTerminal**
2. Enter the command below (cql-to-elm) to translate the modified CQL file. It requires a parameter with the format of file to be translated to (JSON) and the input file to be translated. *Note: The command will save the result file in the same folder as the input file*

```
cql-to-elm --format=JSON --input ~/cql-results/src/data/R4/Statin-Use-Example.cql
```

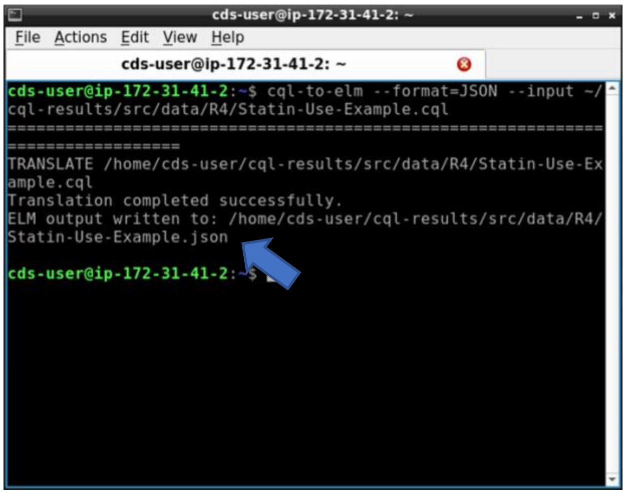

3. Close the **QTerminal**

YOU ARE HERE:

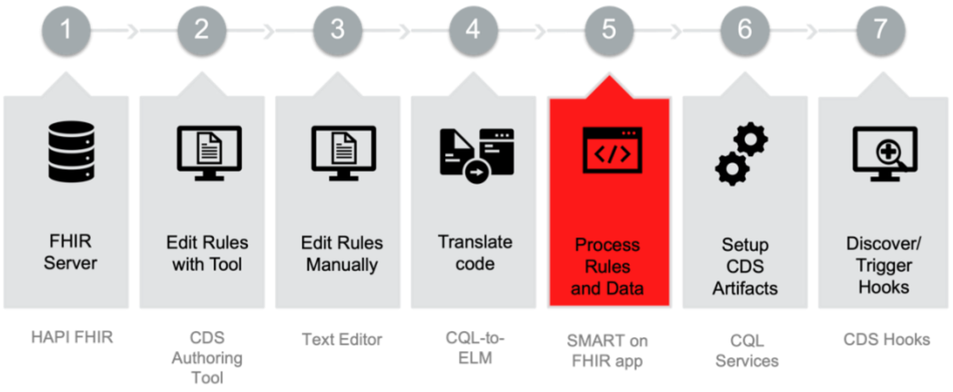

STEP 5: TEST ARTIFACTS

GOAL: In this section, we will launch a prototype SMART on FHIR app (called CQL Results) to test the CQL artifact created above. The app runs a local CQL engine (cql-execution) to process patient data.

> Note that CDS Authoring Tool also has a testing mechanism that allows uploading patient data to test the CQL artifact created. However, we will skip that feature and use a SMART on FHIR app instead.

> You may want to skip this step If…
> - you are not interested in testing the CQL artifacts created
> - you are behind and want to catch up
>
> We have a rescue-files folder in the VM with the final CQL file resulting from this step.
> Go to **Step 6**.

> If you skipped the previous **Step 4**:
> 3. Open the **QTerminal** and type `skip4` to copy the **Statin-Use-Example** (CQL and JSON) files to the folder **cql-results/src/data/R4**
> 4. Close the **Qterminal**

5.1 Launching the CQL Results SMART on FHIR app

1. Click the **CQL Results** bookmark link below the URL bar on the Internet Browser. The launcher will show a list of patients. We include sample patients that can be used to test the CQL artifact created. The synthetic patient data was generated using **Synthea**, a synthetic patient generator developed by the MITRE Corporation.

   Note: The sample patients have a number after their first and last name that was automatically generated by Synthea.
2. The testing patients are:

- **patients for recommendations**
  - Azzie965 Rosenbaum964

- Deangelo7 Kuvalis369
- Eldon28 Spinka232
- Kim439 MacGyver246
- Miles206 Bashirian201

- **patients excluded from the population**
  - Blaine377 Dare640
  - Joseph689 Labadie908
  - Madelaine318 Hessel84
  - Numbers230 Carter549
  - Shanti441 Price929
- **patients not included in the population**
  - Adriana394 Estrada938
  - Cory323 Witting912
  - Elroy493 Pacocha935
  - Kaley842 Reichel38
  - Ricky354 Osinski784

3. Search for the patient **Miles206 Bashirian201** and select it. Enter "Miles" in the search box and press the button **Search**

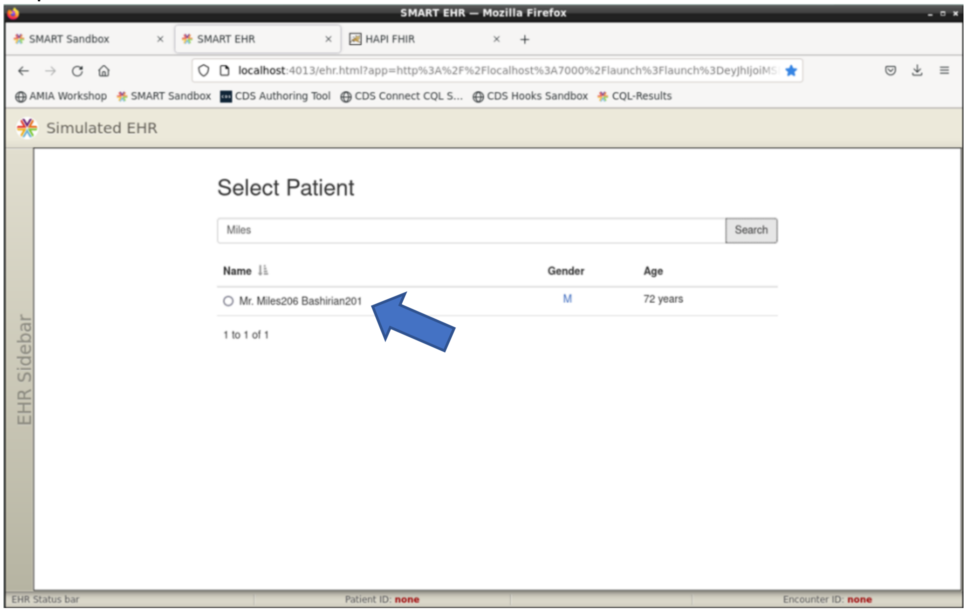

4. The CQL Results will retrieve the patient data and process it against the logic in the default CQL library configured for the app. The default library is displayed on the top right of the app screen (e.g., Condition_and_Medication_Count_FHIRv400).

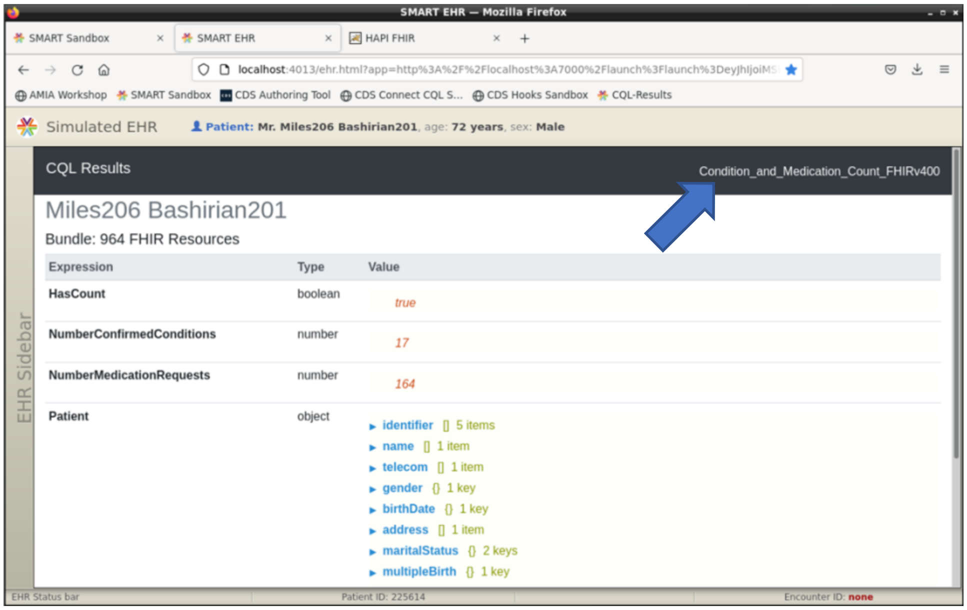

5.2 Loading the Statin Use artifact

5.2.1 Set default CQL library

The app executes the logic of the default CQL Library listed in the **cql-files.json**. Let's set the **Statin-Use-Example** as the new default library. The CQL library parameter is the name of the file containing the CQL library.

1. Open the file manager and navigate to the folder **cql-results/src/data**.
2. Double click the file **cql-files.json**.
3. Replace the current CQL library "**Condition_and_Medication_Count_FHIRv400**" with the new CQL artifact "**Statin-Use-Example**".
   DO NOT include the file extension.
   Note: the library name has first letters capitalized and dashes.
4. Save the file and close the editor window

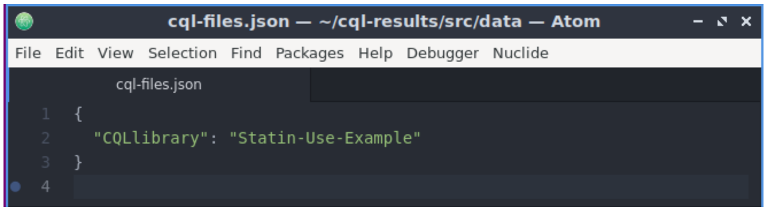

5. The app will automatically reload and process the patient data using the logic of the new CQL artifact. Note that some values are showing as **null**. That's because the artifact uses several value sets and codes from VSAC, which need to be downloaded to be used by the CQL engine. The local value set database is stored in the **valueset-db.json** file (located in the folder **cql-results/src/data**).

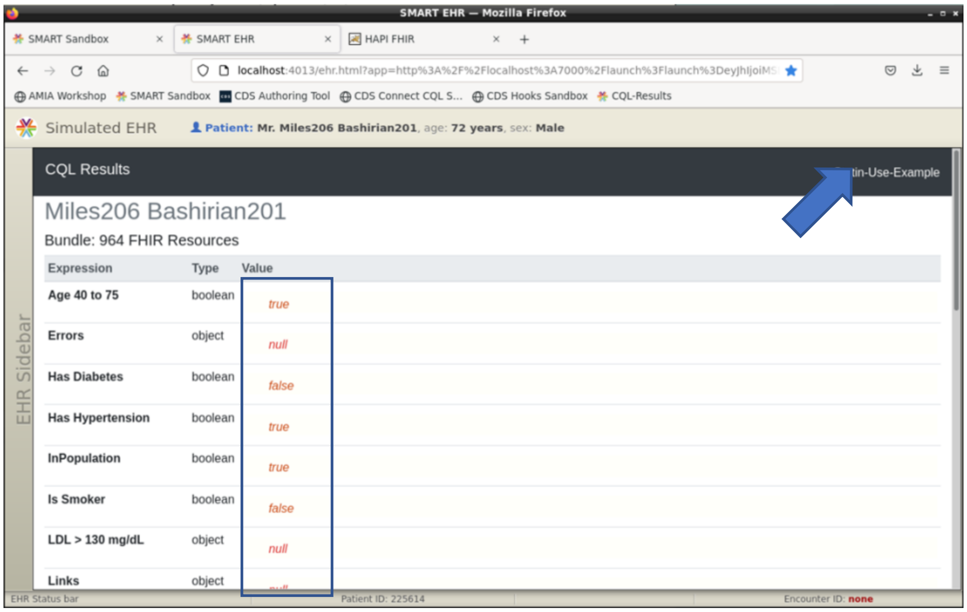

5.2.2 Update the value set database

If you don't have a UMLS account or the account is not setup correctly:

1. Open the **QTerminal** and type `step5.2` to copy the file **valueset-db.json** from the rescue-files folder to the folder **cql-results/src/data** and update the default artifact to **Statin-Use-Example**
2. Close the **QTerminal**

If you have the UMLS account setup correctly, you will update the local value set database with the required VSAC codes. This step will use the UMLS credentials configured at the beginning of the tutorial to automatically login to the NLM Value Set Authority Center (VSAC) and download all value sets and respective codes for all value sets listed in the CQL files. The codes retrieved from VSAC are stored in the file **valueset-db.json** in the folder **cql-results/src/data**.

1. Open the **QTerminal**
2. Enter the command below. The screenshot below shows a message "Loaded 11 value sets". These are all the valuesets available in the CQL artifacts.

```
updateValueset
```

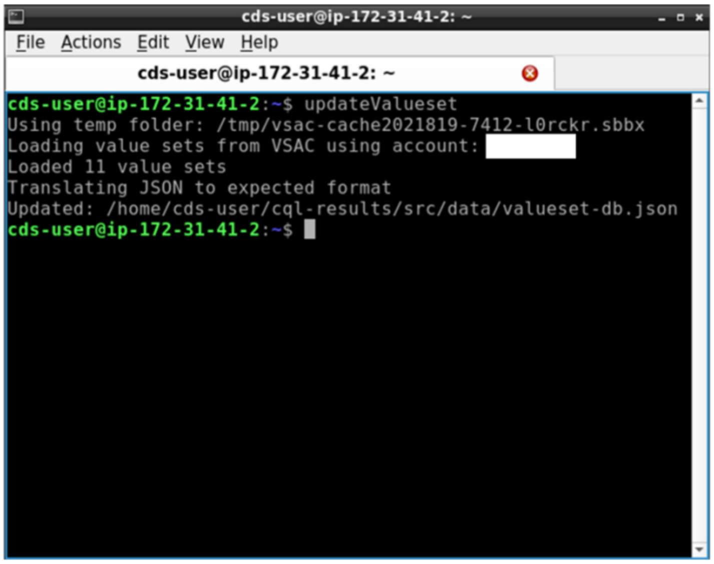

- Close the **QTerminal** window

The app will automatically reload and process the patient data using the CQL logic and the appropriate codes retrieved from VSAC. Note that at this time the ASCVD Risk and other parameters are calculated, and the values are displayed correctly.

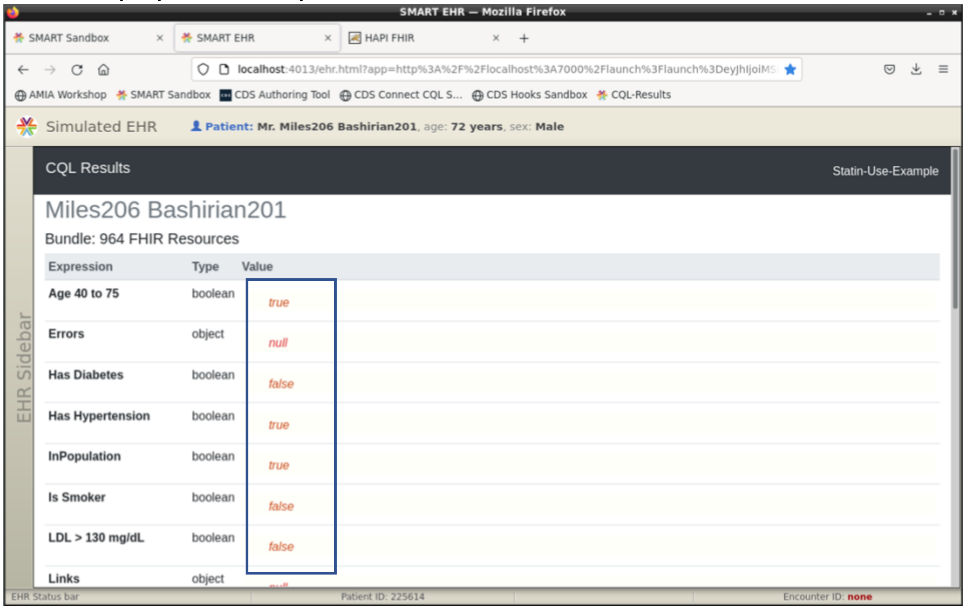

Scroll down to check the value of the CQL statements defined in the artifact. This sample patient would receive a recommendation as shown below.

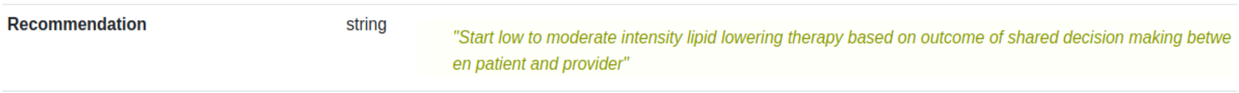

5.3 Select a different patient

1. Click the refresh button of the Internet browser and select a new patient. For example, the patient **Azzie965 Rosenbaum794**.

2. The app will reload and process the new patient data using the same Statin Use artifact.

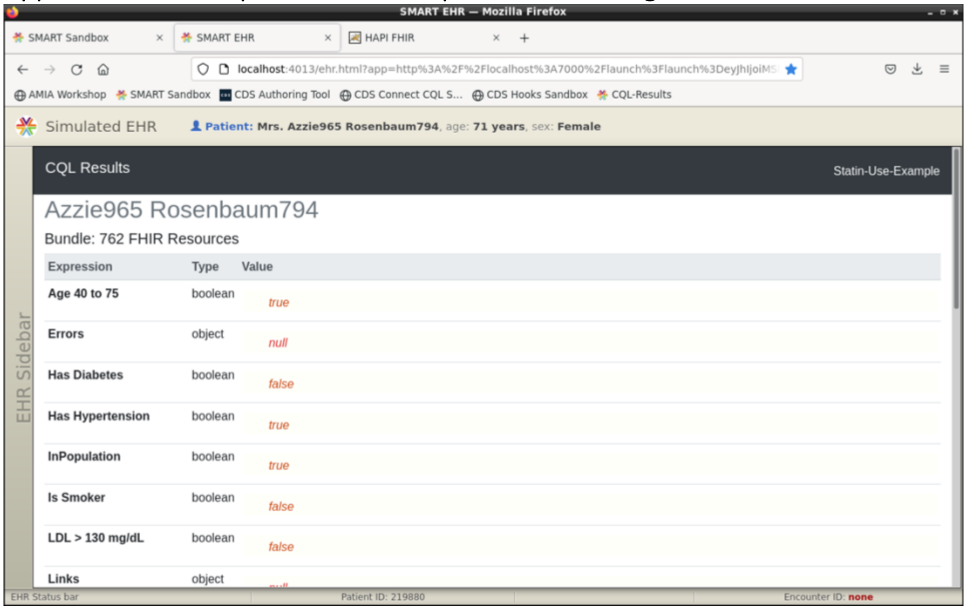

3. Refresh the page in your browser again and switch to patient **Blaine377 Dare640** to see the conditions and the results. Blaine is excluded from the population (Patient meets exclusion criteria) and should not have receive a recommendation.

5.4 Loading the ASCVD Risk artifact

The Statin CDS relies on the calculation of the ASCVD Risk. We can see that Azzie has the 10-year ASCVD Risk >= 10%. The calculation is done by the external CQL imported using the CDS Authoring Tool. Let's change the default CQL library to the external CQL library "**Million_Hearts_Baseline_10_Year_ASCVD_Risk_FHIRv400**" to see the specific risk value and factors used in the calculation.

1. Open Files Manager
2. Navigate to **cql-results/src/data**
3. Double click the file **cql-files.json** and replace the current CQL library "Statin-Use-Example" with the "Million_Hearts_Baseline_10_Year_ASCVD_Risk_FHIRv400"

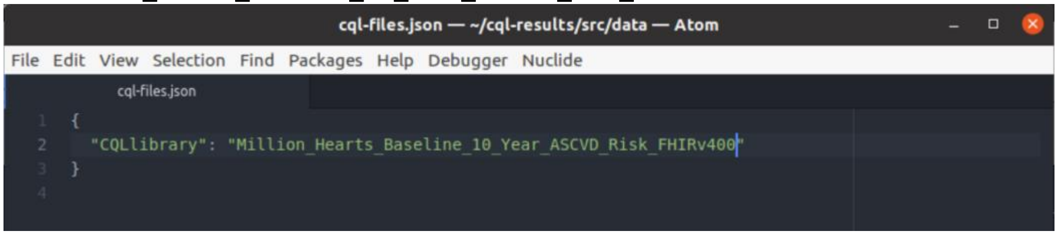

4. Save the file (File -> Save) and close the editor window.
5. Close the Files Manager
6. The app will automatically reload and process the current patient data using the logic of the new default CQL artifact.
7. Switch to another patient to see the result of the ASCV Risk calculation

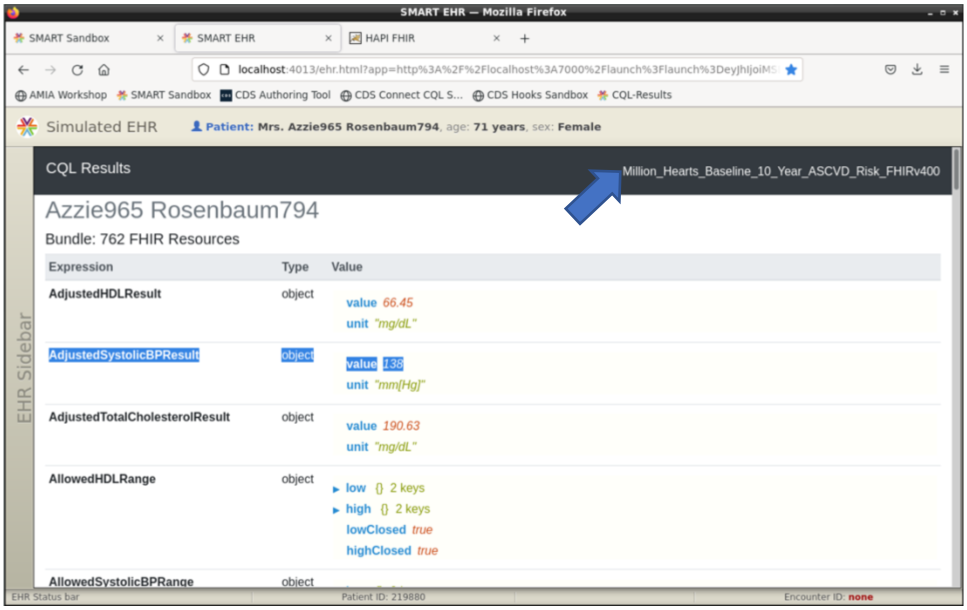

8. Scroll down to check the parameters used to calculate the ASCVD Risk. The ASCVD Risk is represented by the parameter **PatientBaselineRisk**.

YOU ARE HERE:

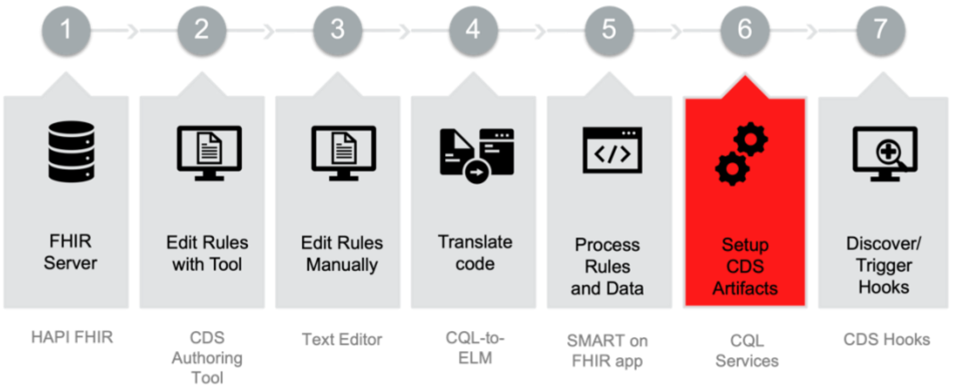

STEP 6: SETUP CQL ARTIFACTS IN CQL SERVICES

GOAL: To make the CQL artifacts available to the CQL Services (CQL Engine) to respond to API requests and CDS Hooks triggering.

The code for the CQL Services was slightly modified to accommodate the needs of this tutorial and to handle a limitation of the CDS Hooks Sandbox in fetching patient data.

You may want to skip this step If…

- you are not interested in writing code manually and you just want to continue the tutorial
- you are behind and want to catch up

We have a rescue-files folder in the VM with the final CQL file resulting from this step. Go to **Step 7**.

If you skipped the previous **Step 5**:

1. Open the **QTerminal** and type `skip5` to setup the default CQL artifact and load the valueset DB file.
2. Close the **Qterminal**

6.1 Stop Docker

1. Open the **QTerminal**
2. Enter the command to stop the CQL Services docker. We will change some parameters and restart it later.

```
docker stop cql-services
```

6.2 Config Libraries

1. In **QTerminal**, type `step6.2` to create a subfolder **statin-use-example-r4** to the folder **AHRQ-CDS-Connect-CQL-SERVICES/config/libraries**. The command will also copy all the necessary CQL

and JSON files supporting the **Statin-Use-Example** artifact. It should include the CQL and JSON of the following files:

a. CDS_Connect_Commons_for_FHIRv400
b. FHIRHelpers
c. CDS_Connect_Conversions
d. Million_Hearts_Baseline_10_Year_ASCVD_Risk_FHIRv400
e. Statin-Use-Example

6.3 Config Hooks

1. In the File Manager, navigate to the folder **AHRQ-CDS-Connect-CQL-SERVICES/config/hooks**
2. Right Click and select **Create New > Blank File**. Name the file as **statin-use-example-r4.json**
   Note: The file name is all lower case and has dashes.
3. Double click the file to open it with the text editor.
4. Copy and paste the content of the Hooks template below into the text editor. The id and title will be displayed on the CQL Services page so that the user knows what Hooks are available. The Hook will use the logic from the Statin-Use-Example artifact (version 0.0.1) created earlier and is configured to be triggered in the patient-view hook. Note that the list of cards is empty. It contains the text `** REPLACE WITH CARDS **`. We will create two cards: one to display the recommendation and one to display a link to a decision aid tool.
   Note: Remove all extra blank lines from the JSON file, if any.

```
{
  "id": "statin-use-example-r4",
  "hook": "patient-view",
  "title": "Statin Use Example for FHIR R4",
  "description": "Presents a United States Preventive Services Task
Force (USPSTF) statin therapy recommendation for adults aged 40 to 75
years without a history of cardiovascular disease (CVD) who have 1 or
more CVD risk factors (i.e., dyslipidemia, diabetes, hypertension, or
smoking) and a calculated 10-year CVD event risk score of 7.5% or
greater.",
  "_config": {
    "cards": [
       ** REPLACE WITH CARDS **
    ],
    "cql": {
      "library": {
        "id": "Statin-Use-Example",
        "version": "0.0.1"
      }
    }
  }
}
```

6.3.1 Card 1: Recommendation

1. Replace the text `** REPLACE WITH CARDS **` with the card below. It will show an information card with the recommendation `${Recommendation}` and a link to the CDS Artifact on the CDS Connect artifact's page. This card will evaluate the condition expression `InPopulation`.

```
{
   "conditionExpression": "InPopulation",
   "card": {
      "summary": "Statin Use for the Primary Prevention of CVD in
Adults",
      "indicator": "info",
      "detail": "${Recommendation}",
      "source": {
         "label": "CDS Connect: Statin Use for the Primary Prevention
of CVD in Adults",
         "url": "https://cds.ahrq.gov/cdsconnect/artifact/statin-use-
primary-prevention-cvd-adults"
      }
   }
}
```

6.3.2 Card 2: Decision Aid Tool

1. Add this new card below the Card 1 in the JSON file. **Note that it starts with a comma to separate the cards.** This card will show a link to the Mayo Clinic's Statin Choice Decision Aid tool.

```
,{
   "conditionExpression": "InPopulation",
   "card": {
      "summary": "Shared Decision Aid Tool for Statin Choice",
      "indicator": "info",
      "detail": "Discuss initiation of low to moderate intensity lipid
lowering therapy",
      "links": [
         {
            "label": "Statin Choice Decision Aid",
            "url": "https://statindecisionaid.mayoclinic.org",
            "type": "absolute"
         }
      ]
   }
}
```

6.3.3 Final CDS Hooks setup file

1. The final content of the hook setup file should look like this. You don't need to copy. This is just to check/compare the content of the file you just created.

```
{
  "id": "statin-use-example-r4",
  "hook": "patient-view",
  "title": "Statin Use Example for FHIR R4",
  "description": "Presents a United States Preventive Services Task
Force (USPSTF) statin therapy recommendation for adults aged 40 to 75
years without a history of cardiovascular disease (CVD) who have 1 or
more CVD risk factors (i.e., dyslipidemia, diabetes, hypertension, or
```

```
smoking) and a calculated 10-year CVD event risk score of 7.5% or
greater.",
  "_config": {
    "cards": [
    {
      "conditionExpression": "InPopulation",
      "card": {
        "summary": "Statin Use for the Primary Prevention of CVD in
Adults",
        "indicator": "info",
        "detail": "${Recommendation}",
        "source": {
          "label": "CDS Connect: Statin Use for the Primary Prevention
of CVD in Adults",
          "url": "https://cds.ahrq.gov/cdsconnect/artifact/statin-use-
primary-prevention-cvd-adults"
        }
      }
    }
    ,{
      "conditionExpression": "InPopulation",
      "card": {
         "summary": "Shared Decision Aid Tool for Statin Choice",
         "indicator": "info",
         "detail": "Discuss initiation of low to moderate intensity
lipid lowering therapy",
         "links": [
         {
            "label": "Statin Choice Decision Aid",
            "url": "https://statindecisionaid.mayoclinic.org/",
            "type": "absolute"
         }
         ]
       }
    }
    ],
    "cql": {
      "library": {
        "id": "Statin-Use-Example",
        "version": "0.0.1"
      }
    }
  }
}
```

2. Save the file (File -> Save)
3. Close the text editor window
4. Close the File Manager

6.4 Restart Docker

1. In the **QTerminal**, enter the command below to start the CQL Services docker.

```
docker start cql-services
```

6.5 Check CQL Services

1. In the internet browser, click the CDS Connect CQL Services bookmark link. The new CDS Hook (**statin-use-exaple-r4**) should be listed on the CQL Services page (see Column Id), and the new services **Statin-Use-Example** and **Million_Hearts_Baseline_10_Year_ASCVD_Risk_FHIRv400** should be listed in the CQL Libraries as shown below.

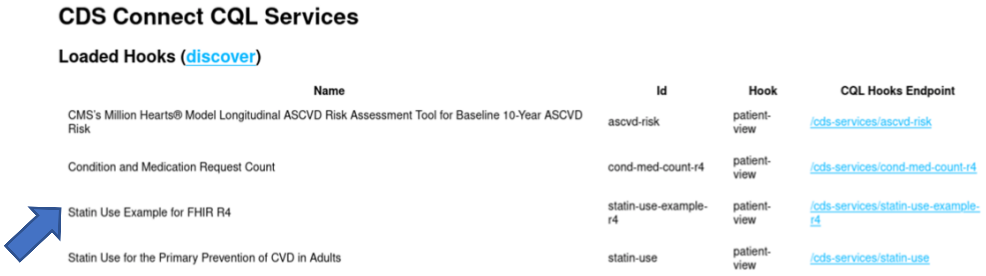
CDS Connect CQL Services

Loaded Hooks (discover)

Name	Id	Hook	CQL Hooks Endpoint
CMS's Million Hearts® Model Longitudinal ASCVD Risk Assessment Tool for Baseline 10-Year ASCVD Risk	ascvd-risk	patient-view	/cds-services/ascvd-risk
Condition and Medication Request Count	cond-med-count-r4	patient-view	/cds-services/cond-med-count-r4
Statin Use Example for FHIR R4	statin-use-example-r4	patient-view	/cds-services/statin-use-example-r4
Statin Use for the Primary Prevention of CVD in Adults	statin-use	patient-view	/cds-services/statin-use

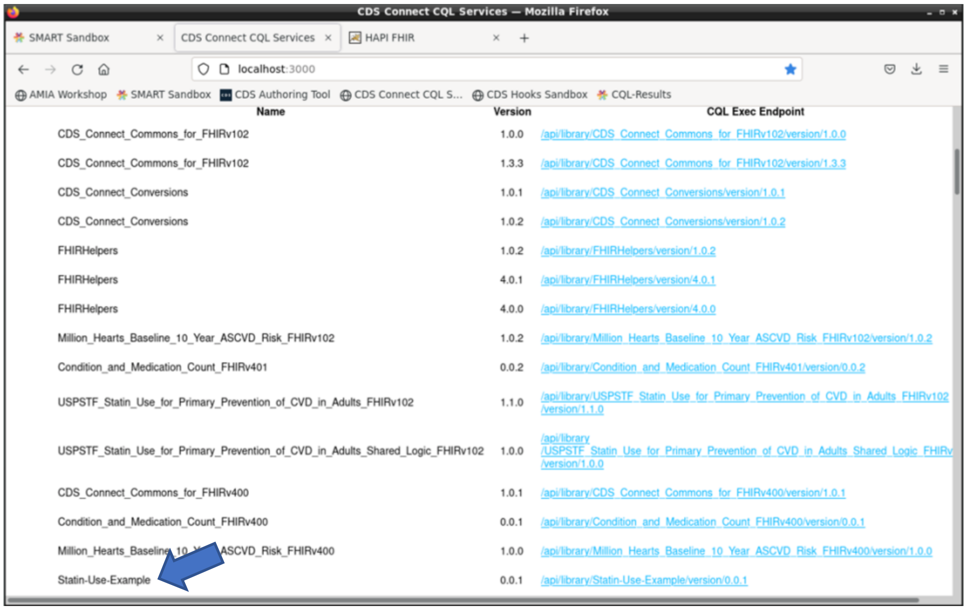

Name	Version	CQL Exec Endpoint
CDS_Connect_Commons_for_FHIRv102	1.0.0	/api/library/CDS_Connect_Commons_for_FHIRv102/version/1.0.0
CDS_Connect_Commons_for_FHIRv102	1.3.3	/api/library/CDS_Connect_Commons_for_FHIRv102/version/1.3.3
CDS_Connect_Conversions	1.0.1	/api/library/CDS_Connect_Conversions/version/1.0.1
CDS_Connect_Conversions	1.0.2	/api/library/CDS_Connect_Conversions/version/1.0.2
FHIRHelpers	1.0.2	/api/library/FHIRHelpers/version/1.0.2
FHIRHelpers	4.0.1	/api/library/FHIRHelpers/version/4.0.1
FHIRHelpers	4.0.0	/api/library/FHIRHelpers/version/4.0.0
Million_Hearts_Baseline_10_Year_ASCVD_Risk_FHIRv102	1.0.2	/api/library/Million_Hearts_Baseline_10_Year_ASCVD_Risk_FHIRv102/version/1.0.2
Condition_and_Medication_Count_FHIRv401	0.0.2	/api/library/Condition_and_Medication_Count_FHIRv401/version/0.0.2
USPSTF_Statin_Use_for_Primary_Prevention_of_CVD_in_Adults_FHIRv102	1.1.0	/api/library/USPSTF_Statin_Use_for_Primary_Prevention_of_CVD_in_Adults_FHIRv102/version/1.1.0
USPSTF_Statin_Use_for_Primary_Prevention_of_CVD_in_Adults_Shared_Logic_FHIRv102	1.0.0	/api/library/USPSTF_Statin_Use_for_Primary_Prevention_of_CVD_in_Adults_Shared_Logic_FHIRv/version/1.0.0
CDS_Connect_Commons_for_FHIRv400	1.0.1	/api/library/CDS_Connect_Commons_for_FHIRv400/version/1.0.1
Condition_and_Medication_Count_FHIRv400	0.0.1	/api/library/Condition_and_Medication_Count_FHIRv400/version/0.0.1
Million_Hearts_Baseline_10_[illegible]ASCVD_Risk_FHIRv400	1.0.0	/api/library/Million_Hearts_Baseline_10_Year_ASCVD_Risk_FHIRv400/version/1.0.0
Statin-Use-Example	0.0.1	/api/library/Statin-Use-Example/version/0.0.1

⚠ If you get a **404 error** or **The connection was reset**, it is likely because the hooks configuration you edited above has problems. Review the steps.

- <u>Potential problem 1</u>: The name of the **statin-use-example-r4** folder created in the libraries folder should be all lower case.
- <u>Potential problem 2</u>: You need the 10 files (CQL and JSON) copied to the **statin-use-example-r4** folder in libraries
- <u>Potential problem 3</u>: The **statin-use-example-r4.json** file created in the hooks folder should be all lower case and the content of the file should not have any blank lines.

You need to fix the CDS Hooks configuration and then stop and start the CQL Services again.

2. Scroll down to check the list of returnable expressions from the new Statin-Use-Example artifact. These are the same expressions displayed by the CQL Results app.

YOU ARE HERE:

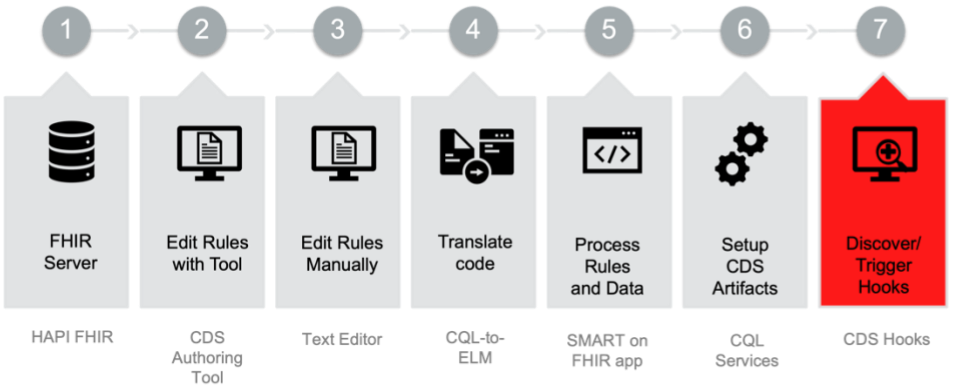

STEP 7: TEST CDS HOOKS

GOAL: To test the triggering of a Hook that uses the CQL artifact created. We will use the CDS Hooks Sandbox which provides an EHR simulator that can trigger hooks at patient-view.

If you skipped the previous **Step 6**:

3. Open the **QTerminal** and type `skip6` to setup the CDS-Hooks configuration files.
4. Close the **QTerminal**

7.1 Discover Services

1. Click the **CQL Services** bookmark link below the URL bar on the internet browser.
2. Right click the link **discover** and select the option **Copy Link**. The link is http://localhost:3000/cds-services.

Loaded Hooks (discover)

7.2 Setup Hook

1. Open the **CDS Hooks Sandbox** bookmark link below the URL bar on the Internet browser. It will show the default greetings card for patient Abdul Koepp from our local FHIR Server.

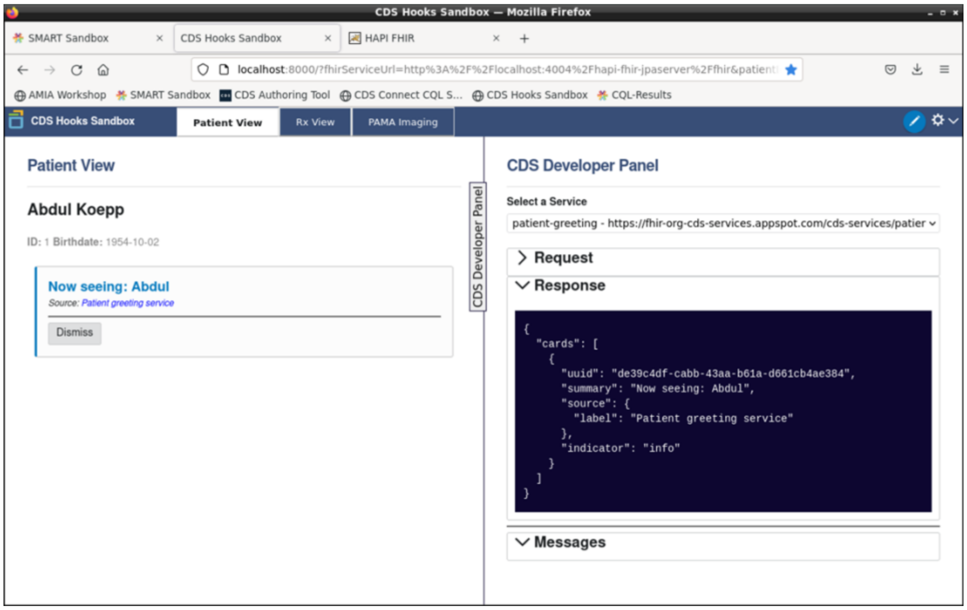

2. Click the gear icon on the top right and select the option **Add CDS Services**
3. Paste the link to discover CDS services (you may need to right click to paste)

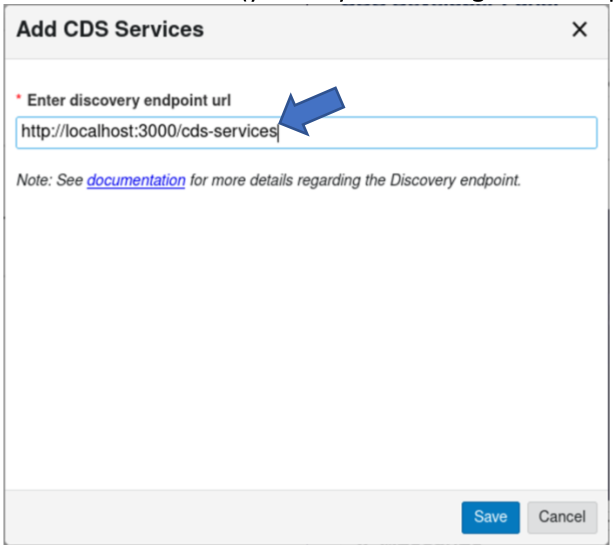

4. Click the button **Save**.
5. The system will add the new service and will trigger the Condition and MedicationRequest Count card on the left-hand side. The statin use is not triggered because the patient does not meet the inclusion criteria.

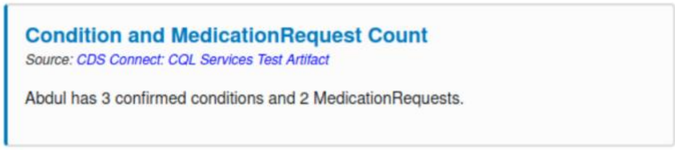

7.3 Setup Patient

1. Let's change to a different patient from our list of testing patients.

2. Click the gear icon on the top right again and select the option **Change Patient**
3. Enter the ID **225614** for patient Miles

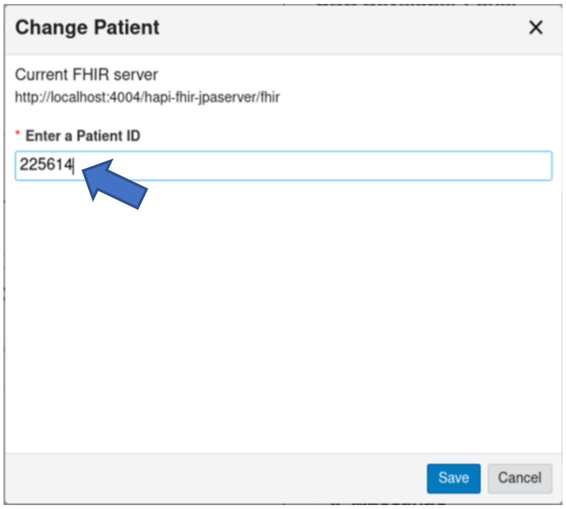

4. Click on the **Save** button
5. The system should trigger the Statin-use hook as Miles receives the recommendation
   NOTE: It may take several seconds for the Statin Use card to appear
6. Change the Selected Service on the right panel to **statin-use-example-r4**. After 15-20 seconds, it will show the content of the response cards generated by the CQL Services, including the link to the Statin Choice Decision Aid tool.

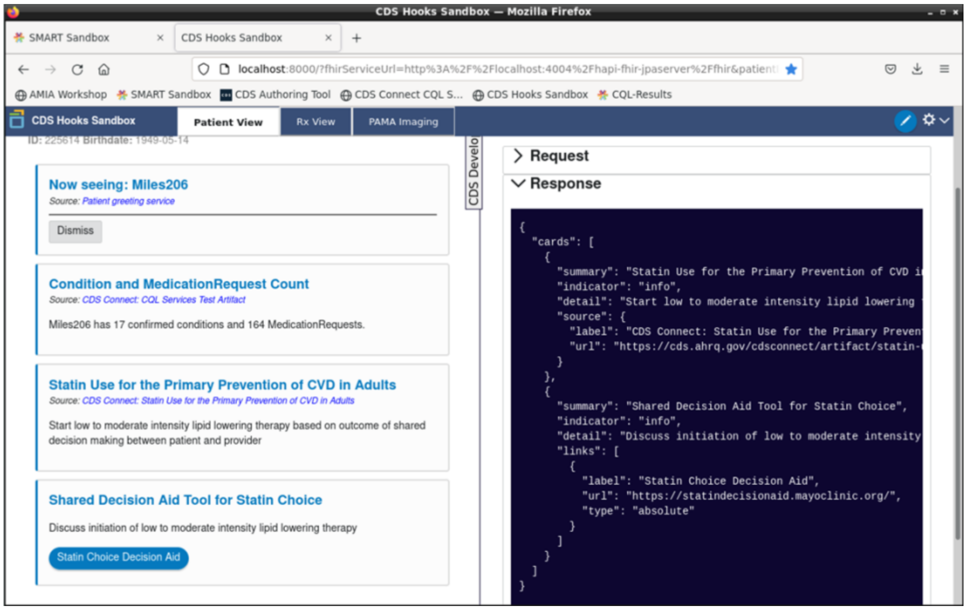

7. Repeat the process with different patients to see the results for patients included and excluded from the population. Note that when you switch patients, the CDS Hooks sandbox does not clear previous cards right away, so it may take 10 – 15 seconds for all cards to change or disappear when switching patients. Blaine and Ricky will not result in any cards being returned.

   a. patients for recommendations: Azzie Rosenbaum (ID = 219880)

b. patients excluded from the population: Blaine Dare (ID = 220642)
c. patients not included in the population: Ricky Osinski (ID = 227577)

If you get an error or the cards don't trigger, it might be a configuration error for the CDS Hooks. To address this issue, replace the file **satin-use-example-r4.json** and try again. You will need to stop and start the cql-services again.

Congratulations!

You completed the tutorial.

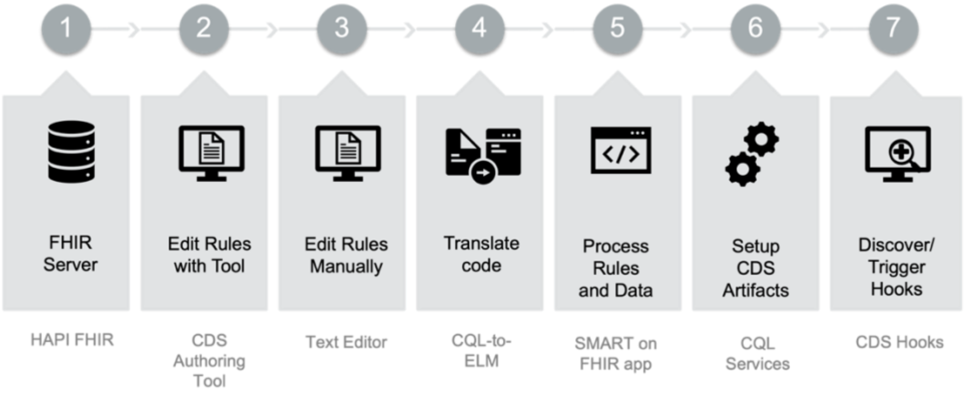

Supplement: ooad038_Supplementary_Data [file ooad038_supplementary_data.pdf]
